# Vascular niche IL-6 induces alternative macrophage activation in glioblastoma through HIF-2α

**DOI:** 10.1038/s41467-018-03050-0

**Published:** 2018-02-08

**Authors:** Qirui Wang, Zhenqiang He, Menggui Huang, Tianrun Liu, Yanling Wang, Haineng Xu, Hao Duan, Peihong Ma, Lin Zhang, Scott S. Zamvil, Juan Hidalgo, Zhenfeng Zhang, Donald M. O’Rourke, Nadia Dahmane, Steven Brem, Yonggao Mou, Yanqing Gong, Yi Fan

**Affiliations:** 1 Department of Radiation Oncology, University of Pennsylvania Perelman School of Medicine, Philadelphia, PA 19104 USA; 2 School of Traditional Chinese Medicine, Southern Medical University, 510515 Guangzhou, China; 3 Department of Neurosurgery, Sun Yat-sen University Cancer Center, 510060 Guangzhou, China; 4 Department of Otorhinolaryngology, Division of Head and Neck Surgery, The Sixth Affiliated Hospital of Sun Yat-sen University, 510655 Guangzhou, China; 5 Department of Obstetrics and Gynecology, University of Pennsylvania Perelman School of Medicine, Philadelphia, PA 19104 USA; 6 Department of Neurology and Program in Immunology, University of California at San Francisco, San Francisco, CA 94158 USA; 7 Department of Cellular Biology, Physiology, and Immunology, Autonomous University of Barcelona, 08193 Barcelona, Spain; 8 Department of Radiology The, Second Affiliated Hospital of Guangzhou Medical University, 510260 Guangzhou, China; 9 Department of Neurosurgery, University of Pennsylvania Perelman School of Medicine, Philadelphia, PA 19104 USA; 10 Department of Medicine, Division of Human Genetics and Translational Medicine, University of Pennsylvania Perelman School of Medicine, Philadelphia, PA 19104 USA

## Abstract

Spatiotemporal regulation of tumor immunity remains largely unexplored. Here we identify a vascular niche that controls alternative macrophage activation in glioblastoma (GBM). We show that tumor-promoting macrophages are spatially proximate to GBM-associated endothelial cells (ECs), permissive for angiocrine-induced macrophage polarization. We identify ECs as one of the major sources for interleukin-6 (IL-6) expression in GBM microenvironment. Furthermore, we reveal that colony-stimulating factor-1 and angiocrine IL-6 induce robust arginase-1 expression and macrophage alternative activation, mediated through peroxisome proliferator-activated receptor-γ-dependent transcriptional activation of hypoxia-inducible factor-2α. Finally, utilizing a genetic murine GBM model, we show that EC-specific knockout of IL-6 inhibits macrophage alternative activation and improves survival in the GBM-bearing mice. These findings illustrate a vascular niche-dependent mechanism for alternative macrophage activation and cancer progression, and suggest that targeting endothelial IL-6 may offer a selective and efficient therapeutic strategy for GBM, and possibly other solid malignant tumors.

## Abstract (web summary)

Macrophages in the tumour microenvironment (TME) acquire tumour-promoting functions upon M2 polarization. Here the authors show, in a mouse model of glioblastoma, that endothelial cells in the TME induce macrophage M2 polarization via IL-6 and that depletion of endothelial IL-6 improves survival.

## Introduction

Most malignant solid tumors are characterized by extensive infiltration of inflammatory leukocytes. Among them, tumor-associated macrophages play a pivotal role in tumor growth, cancer immunosuppression, and therapy resistance^1–3^. In contrast to classically activated macrophages that stimulate phagocytosis, inflammation, and host immunity, a prominent population of macrophages in tumor microenvironment undergoes alternative activation to acquire tumor-promoting functions, for example, these macrophages express anti-inflammatory cytokines, such as interleukin-10 (IL-10), and tumor growth factor-β (TGF-β), and arginase-1 that inhibits nitric oxide (NO) production and produces ornithine^4–7^. Growing evidence suggests that alternative macrophage activation is a driving force that fuels cancer progression, but the underlying tumor microenvironment-dependent mechanisms remain largely unknown.

Glioblastoma multiforme (GBM), the grade IV glioma, is the most common and most aggressive primary brain tumor. GBM is among the most lethal of human malignancies, with a current median overall survival of approximately 14 months^8, 9^, largely due to its high resistance to standard-of-care treatments including surgical resection, radiation, and chemotherapy^10^. The development of new therapies is therefore urgently needed, in which targeting tumor immunity holds great promise for GBM treatment. Notably, macrophages are a major population of the non-neoplastic cells in GBM, evidenced by as many as half of the cells in GBM tumors are macrophages or microglia^11, 12^, suggesting that tumor-associated macrophages may represent an indispensable target for immunotherapy. Likewise, a recent study shows that receptor inhibition of colony-stimulating factor-1 (CSF-1), a major factor for macrophage differentiation and survival, alters alternative macrophage polarization and blocks GBM progression^13^.

A multitude of evidence shows that macrophages stimulate glioma growth and invasion and induce therapeutic resistance^12, 14^. Glioma-associated macrophages express and secrete multiple factors including STI1, EGF (epidermal growth factor), TGF-β, and MT1-MMP to promote glioma cell survival, proliferation, and migration^15–19^. On the other hand, glioma cells induce macrophage recruitment by releasing chemoattractants CXCL12, GDNF, and CSF-1^19–21^. However, how macrophage activation is spatiotemporally regulated in glioma is largely unclear, which is critical for the development of new therapies against GBM. Here, we reveal a vascular niche-dependent regulatory system for macrophage activation, targeting which may offer new therapeutic opportunities for the treatment of GBM, and possibly other solid malignant tumors.

## Results

### Vasculature-associated alternative macrophage activation

**Fig. 1:**
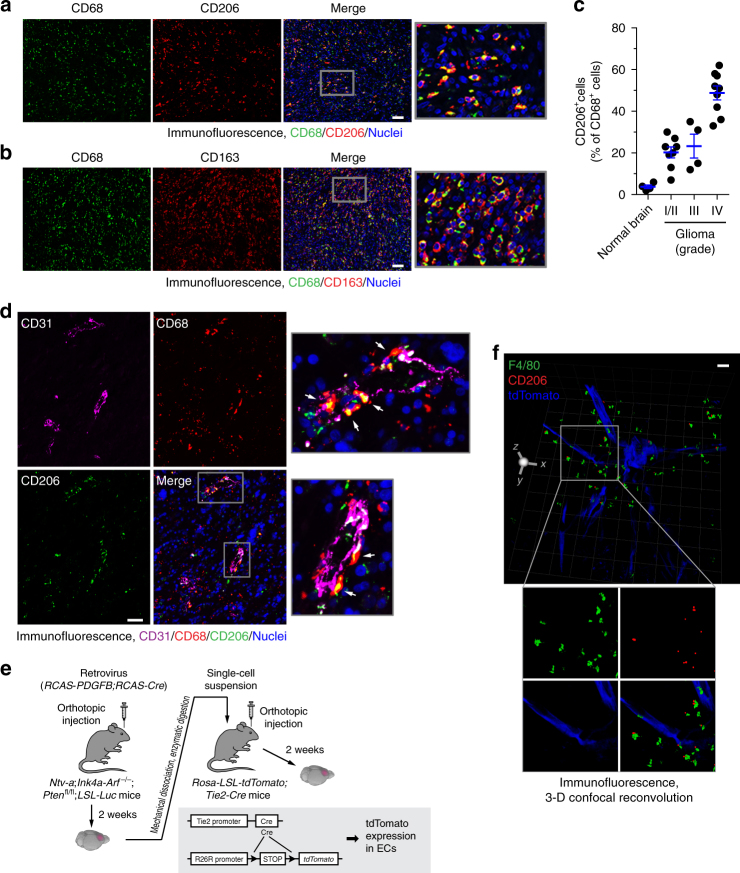
Alternatively activated macrophages are localized proximately to GBM-associated ECs. **a**–**d** Tissue sections from human normal brain and surgical specimens of human glioma tumors were probed with different antibodies. **a** GBM tumor sections were stained with anti-CD68 and anti-CD206 antibodies. Representative images are shown (*n* = 5 GBM patient tumors). Bar represents 100 μm. Zoom-in factor: 4. **b** GBM tumor sections were stained with anti-CD68 and anti-CD163 antibodies. Representative images are shown (*n* = 5 patient GBM tumors). Bar represents 100 μm. Zoom-in factor: 4. **c** Normal brain and GBM tumor sections were stained with anti-CD68 and anti-CD206 antibodies. Quantified data are shown (total *n* = 4 normal brains and 21 glioma tumors, mean ± SEM). **d** GBM tumor sections were stained with anti-CD31, anti-CD206, and anti-CD68 antibodies. Representative images are shown (*n* = 5 patient GBM tumors). Arrows indicate CD68^+^CD206^+^ cells. Bar represents 100 μm. Zoom-in factor: 4. **e**, **f** GBM was induced by RCAS-mediated gene transfer in *Ntv-a;Ink4a-Arf*^−/−^;*Pten*^fl/fl^;*LSL-Luc* mice, followed by orthotopic tumor transplantation into *Rosa-LSL-tdTomato*;*Tie2-Cre* mice. **e** Experimental procedure. **f** Thick sections were stained with anti-F4/80 and anti-CD206 antibodies, and subjected to confocal scanning imaging. 3-D images were generated and shown. Bar represents 200 μm. Zoom-in factor: 1.6

We investigated potential alternative macrophage activation in human GBM tumors. Although there are currently no specific surface markers identified for distinct macrophage activation, alternatively activated macrophages reliably express CD206 and CD163 (and anti-inflammatory cytokine IL-10), in contrast to the expression of CD86 (and proinflammatory cytokine IL-12) by classically activated macrophages^4, 22^. Immunofluorescence analysis of surgical tumor specimens from human patients with different grades of gliomas showed that a large population of GBM-associated CD68^+^ macrophages robustly expressed CD206 and CD163 (Fig. 1a, b) and relatively expressed CD86 at a lower level (Supplementary Fig. 1), while only small population of CD68^+^ macrophages or microglia cells expressed CD206 in normal brains (Supplementary Fig. 1). Moreover, consistent with previously published work showing that glioma grades correlate with the expression of multiple alternative activation markers in tumor-associated macrophages^23^, there was an increase in CD206 expression by tumor-associated macrophages from different grades of gliomas (Fig. 1c), suggesting enhanced alternative activation in these macrophages. As a critical marker for the anti-inflammatory macrophage subset, arginase-1 competes with inducible nitric oxide synthase (iNOS) and hydrolyzes l-arginine into urea and ornithine, a precursor to l-proline and polyamines, which suppress NO-mediated cytotoxicity via l-arginine consumption, enhance collagen synthesis and fibrosis via l-ornithine formation, and increase cellular proliferation via polyamine generation, all important for macrophage-mediated tumor-promoting functions^24, 25^. Our data indicate that a majority of GBM-associated macrophages expressed arginase-1 (Supplementary Fig. 2), verifying the increased alternative activation of macrophages in GBM.

Interestingly, immunofluorescence analysis of these human specimens indicated that CD206^+^CD68^+^ macrophages were localized proximately to CD31^+^ vascular endothelial cells (ECs) (Fig. 1d). To spatially precisely analyze their distribution, we took advantage of a fluorescence protein-based genetic labeling system to visualize the vasculature, in which tdTomato expression is driven by EC-specific Tie2 promoter in mice (Fig. 1e). An orthotopic, genetic murine GBM model with a native microenvironment was induced by *RCAS*/*N-tva*-mediated somatic PDGF gene transfer in *Ink4a-Arf*^−/−^;*Pten*^−/−^ neural stem/progenitor cells, followed by tumor transplantation into the *Tie2*-*Cre*;*ROSA-LSL-tdTomato* mice (Fig. 1f). Consistent with the observations in human subjects, three-dimensional reconvolution of confocal scanning images showed that F4/80^+^CD206^+^ macrophages were localized near the tdTomato^+^ ECs (Fig. 1f and Supplementary Movie 1), implicating a possible role of tumor-associated ECs in alternative macrophage activation in GBM.

### Glioma ECs promote alternative macrophage polarization

**Fig. 2:**
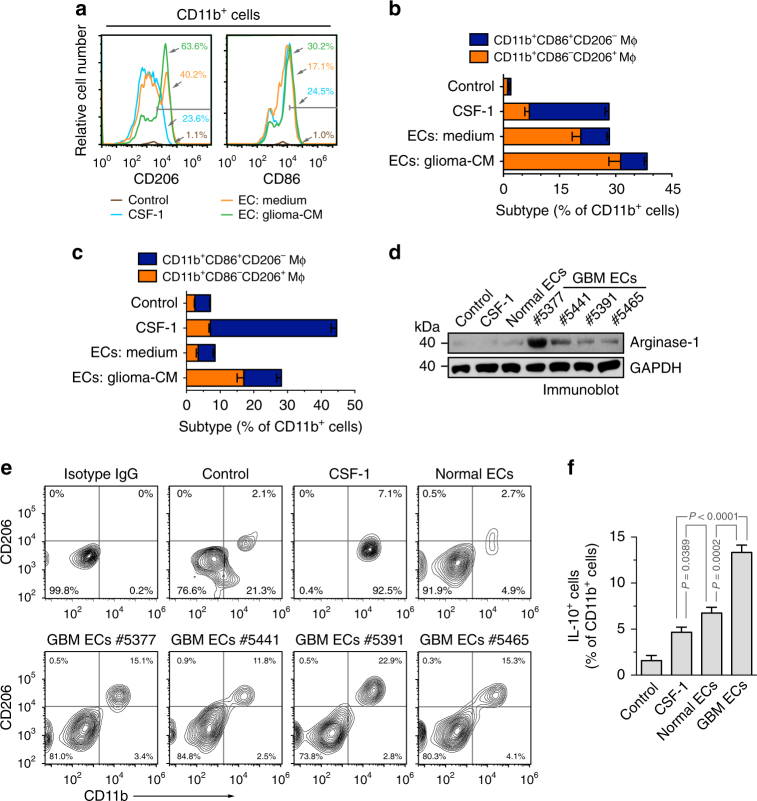
GBM ECs induce alternative activation of macrophages. **a**,** b** Mouse brain microvascular ECs were pretreated with the glioma-conditioned medium (glioma-CM, harvested from medium supernatant of mouse GL26 glioma cells under 1% hypoxia) or control medium for 24 h. Mouse bone marrow (BM)-derived macrophages were incubated with CSF-1 or co-cultured with pretreated ECs for 5 days, stained with anti-CD11b, anti-CD86, anti-CD206 antibodies, and analyzed by flow cytometry. **a** Representative results of CD206 and CD86 expression in CD11b^+^ cells. **b** Quantified data in sorted CD11b^+^ macrophages (Mϕ, *n* = 3–4 mice, mean ± SEM). **c** Human brain microvascular ECs were pretreated with the glioma-CM (harvested from medium supernatant of human U251 glioma cells under 1% hypoxia) or control medium for 24 h. Human peripheral blood mononuclear cell (PBMC)-derived monocytes were incubated with CSF-1 or co-cultured with pretreated ECs for 5 days, stained with anti-CD11b, anti-CD86, anti-CD206 antibodies, and subjected to flow cytometry analysis. Quantified data in sorted CD11b^+^ cells (*n* = 3, mean ± SEM). **d** Human PBMC-derived monocytes were incubated for 5 days with CSF-1, or co-cultured with tumor-associated ECs isolated from different GBM patients or human normal brain microvascular ECs in upper and lower chambers of transwells, respectively. Monocytes were harvested and subjected to immunoblot analysis with anti-arginase-1 and anti-GAPDH antibodies. **e** Human PBMC-derived monocytes were incubated for 5 days with CSF-1, or co-cultured with tumor-associated ECs isolated from different GBM tumors (*n* = 4 patients) or human normal brain microvascular ECs. Cells were harvested, stained with anti-CD206, and anti-CD86 antibodies, and subjected to flow cytometry analysis. Representative images are shown. **f** PBMC-derived monocytes were incubated for 5 days with CSF-1, or co-cultured with tumor-associated ECs isolated from GBM patient #5377 or human normal brain microvascular ECs. Cells were harvested, stained with anti-IL-10 and anti-CD11b antibodies, and subjected to flow cytometry analysis (*n* = 5, mean ± SEM). *P* values were determined by Student’s *t* test

To test the role of tumor-associated ECs in macrophage alternative activation, we pretreated mouse ECs with the conditioned medium (CM) harvested from the medium supernatant of cultured mouse GL26 glioma cells, and co-cultured these ECs with mouse bone marrow (BM) cells. CD11b^+^ macrophages were analyzed by flow cytometry for macrophage activation. Our data revealed that co-culture with control ECs, and to a greater extent, with glioma-CM-pretreated ECs, induced robust CD206 expression: notably, over 60% of CD11b^+^ macrophages positively expressed CD206 when co-cultured with glioma-CM-pretreated ECs, implicating that glioma microenvironment-stimulated ECs promote alternative activation of macrophage (Fig. 2a). In contrast, both CSF-1 treatment and co-culture with ECs induced comparable CD86 expression in CD11b^+^ macrophages. Additionally, the robust CD206 expression in macrophages induced by co-cultured glioma-CM-pretreated ECs was validated by immunofluorescence (Supplementary Fig. 3). To specifically analyze classically and alternatively activated macrophages, we sorted CD11b^+^CD86^+^CD206^−^ and CD11b^+^CD86^−^CD206^+^, respectively. Our data showed that glioma-CM-pretreated ECs trend to induce alternative macrophage activation (Fig. 2b). To verify these results in human subjects, we pretreated human brain ECs with the CM harvested from the medium supernatant of cultured human GBM cells, and co-cultured these cells with human peripheral blood mononuclear cell (PBMC)-derived monocytes, followed by flow cytometry analysis. Consistent with the results from mouse studies, we showed that glioma-CM-pretreated human ECs stimulated human monocytes toward alternative activation, as evidenced by increased CD206^+^CD86^+^ populations in CD11b^+^ cells (Fig. 2c). In addition, our data showed that glioma-CM alone slightly induced CD206 expression and macrophage alternative activation in human monocytes under normoxia, but moderately stimulated CD206 expression and alternative activation under hypoxia (Supplementary Fig. 4), implicating a possible role of hypoxia in macrophage polarization. However, hypoxia slightly promoted macrophage alternative activation but did not further enhance tumor EC-induced CD206 expression and alternative activation (Supplementary Fig. 5).

Moreover, PBMC-derived monocytes were co-cultured in transwells with normal brain ECs or GBM-associated ECs that were isolated from human GBM specimens. Immunoblot analysis shows that co-culture with GBM ECs induced robust arginase-1 expression in human monocytes (Fig. 2d), validating the stimulatory effects of tumor-associated ECs on alternative polarization of macrophages. Furthermore, PBMC-derived monocytes were directly co-cultured with brain ECs or GBM-associated ECs. Flow cytometry analysis showed that co-culture with GBM-derived ECs, but not normal ECs or CSF-1, induced robust CD206 expression in CD11b^+^ macrophages (Fig. 2e). Finally, we analyzed anti-inflammatory cytokine IL-10 expression in these treated monocytes, as a function readout for alternative macrophage activation. Our data showed that co-culture with GBM ECs remarkably enhanced IL-10 expression in human monocytes (Fig. 2f), further verifying EC-mediated alternative macrophage activation in GBM. Taken together, these findings suggest a vascular niche for alternative polarization of macrophages in glioma microenvironment.

### EC-secreted IL-6 induces macrophage alternative activation

**Fig. 3:**
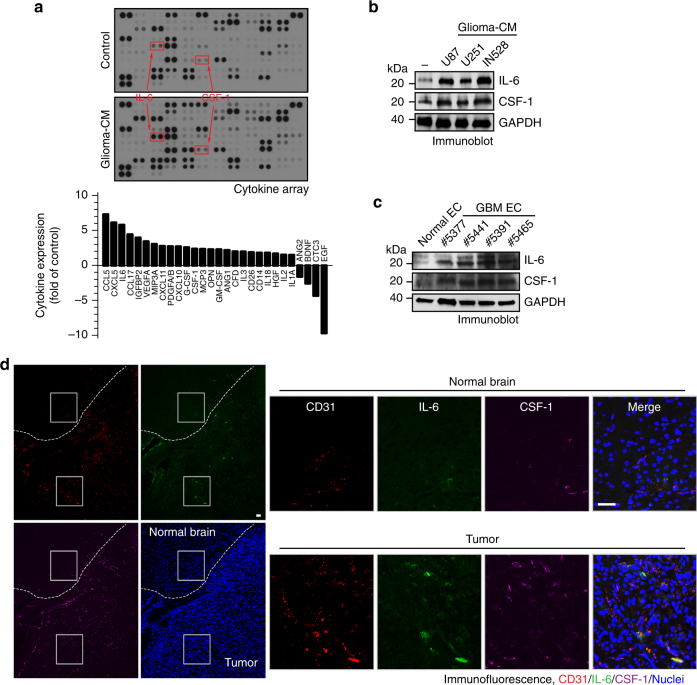
GBM ECs express IL-6. **a** Human brain ECs were treated with glioma-CM for 24 h, and cell lysates were subjected to multiplex cytokine array analysis. Left, a representative blot. Right, quantified dot intensity of most significantly changed cytokines. **b** Human microvascular brain ECs were treated with glioma-CM that were harvested from different human glioma cells. Cell lysates were immunoblotted. **c** Human microvascular brain ECs and tumor-associated ECs isolated from different GBM patients were subjected to immunoblot analysis. **d** Mouse GBM was induced by orthotopic injection of GL26 glioma cells into wild-type mouse. The brain sections that include normal brains and tumors were stained with anti-CD31, anti-IL-6, and anti-CSF-1 antibodies. Representative immunofluorescence images are shown. Right, enlarged area in normal and tumor tissues. Bar represents 50 μm. Zoom-in factor: 4

We next investigated the mechanism(s) by which GBM-associated ECs induce macrophage alternative activation, initially focusing on EC secretion. Multiplex cytokine array analysis of medium supernatants indicated that glioma-CM remarkably altered EC expression of multiple cytokines and growth factors (Fig. 3a). Most robustly up-regulated cytokines included CCL5 and CXCL5, which regulate macrophage chemotaxis, and IL-6 and CSF-1, which have known functions acting on macrophages, particularly considering a recently published work showing a role of IL-6 in macrophage alternative activation in diabetic inflammation^26^. Our data showed that CCL5 and CXCL5 did not induce CD206 expression or macrophage alternative activation (Supplementary Fig. 6). Immunoblot analysis confirmed that glioma-CM remarkedly increased IL-6 expression and moderately increased CSF-1 expression in ECs (Fig. 3b), and also showed that GBM-associated ECs constitutively expressed IL-6 and CSF-1 at a higher level than normal brain ECs (Fig. 3c). Consistently with these in vitro results, our in vivo study with an orthotopic, syngenetic GL26 glioma model indicated a remarked increase in IL-6 expression by tumor-associated ECs, in comparison to normal brain ECs (Fig. 3d). Interestingly, IL-6, but not CSF-1, was preferentially localized in CD31^+^ ECs, implying that ECs as a major source for the expression of IL-6 but not CSF-1 in glioma microenvironment.

**Fig. 4:**
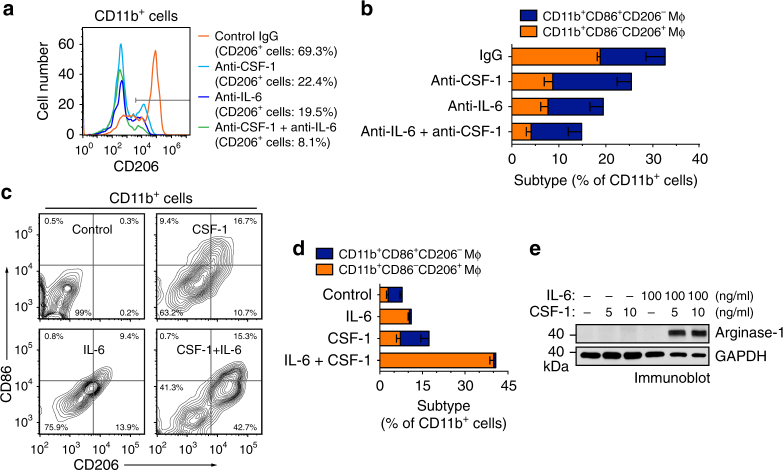
IL-6 is critical for EC-induced macrophage alternative activation. **a**, **b** Mouse microvascular brain ECs were pretreated with the glioma-CM for 24 h. Mouse BM-derived macrophages were co-cultured with pretreated ECs for 5 days in the presence of control IgG, anti-CSF-1 antibody, or anti-IL-6 antibody or both antibodies. The cells were stained with anti-CD11b, anti-CD86, anti-CD206 antibodies, and analyzed by flow cytometry. **a** Representative sorting for CD206 expression in CD11b^+^ cells. **b** Quantified data in sorted CD11b^+^ cells (*n* = 3–5, mean ± SEM). **c**–**e** Mouse BM-derived macrophages were treated with IL-6 and CSF-1 for 5 days. **c**, **d** The cells were stained with anti-CD11b, anti-CD86, and anti-CD206 antibodies, and analyzed by flow cytometry. **c** Representative sorting for CD206 and CD86 expression in CD11b^+^ cells. **d** quantified data in sorted CD11b^+^ cells (*n* = 3, mean ± SEM). **e** Cell lysates were immunoblotted

We next tested the role of IL-6 in EC-induced macrophage alternative activation. Our data showed that both IL-6-neutralizing and CSF-1-neutralizing antibodies reduced CD206^+^ cell population in CD11b^+^ mouse BM macrophages treated with glioma-CM-pretreated ECs, but did not affect CD11b^+^CD86^+^CD206^−^ population (Fig. 4a, b), suggesting a crucial role of IL-6 and CSF-1 in EC-induced alternative macrophage activation. Consistent with this finding, small interfering RNA (siRNA)-mediated knockdown of IL-6 in ECs inhibited EC-induced arginase-1 expression (Supplementary Fig. 7). Interestingly, treatment of mouse BM-derived macrophages (BMDMs) with purified IL-6 moderately stimulated CD206 expression, but the combined treatment with IL-6 and CSF-1 induced alternative polarization of macrophages, as evidenced by robustly enhanced CD206^+^ population in CD11b^+^ cells (Fig. 4c, d). Consistently, the combined treatment with IL-6 and CSF-1 increased CD206 population in human PBMC-derived monocytes (Supplementary Fig. 8). Furthermore, combined treatment with IL-6 and CSF-1, but not single treatment, remarkably induced arginase-1 expression (Fig. 4e). Together, these results suggest a role of EC-secreted IL-6, and possibly microenvironmental CSF-1, for macrophage alternative activation in GBM.

### CSF-1 and IL-6 induce macrophage polarization through HIF-2α

**Fig. 5:**
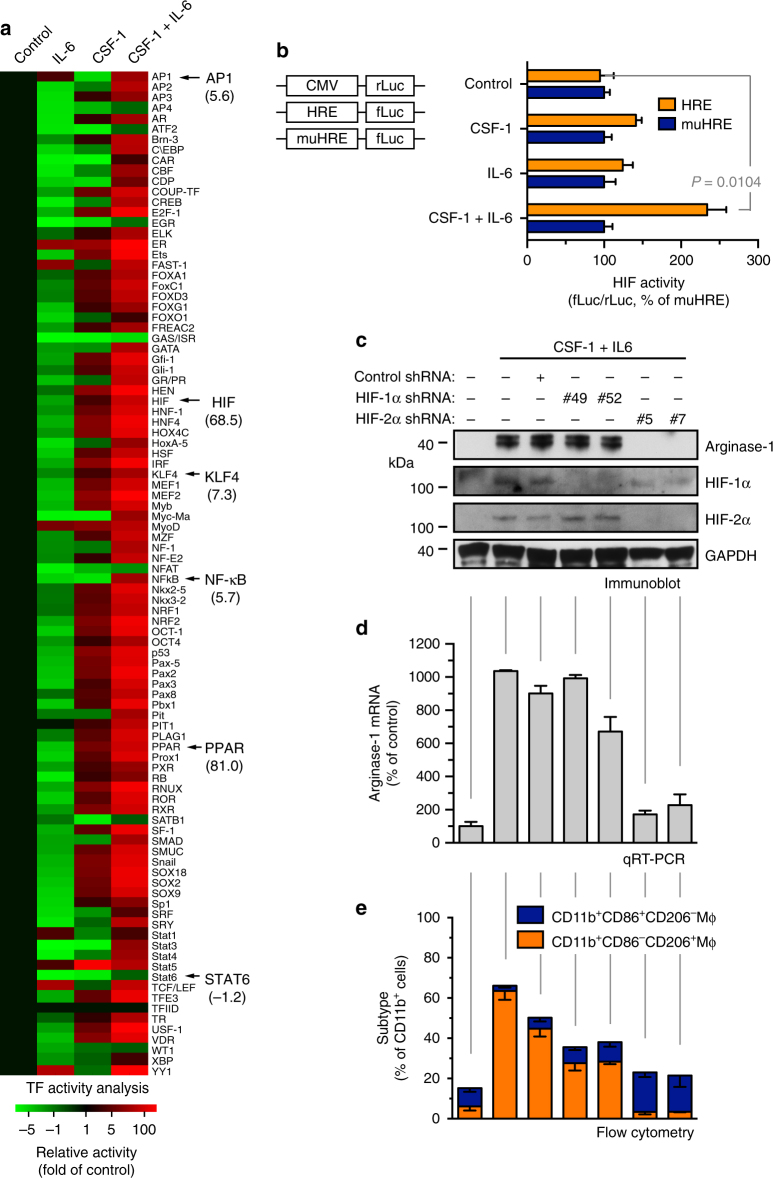
HIF-2α is critical for IL-6-mediated arginase-1 expression and alternative macrophage activation. **a** Mouse BM-derived macrophages were treated with CSF-1 and IL-6 for 3 days. Nuclei proteins were subjected to multiplex profiling analysis for transcriptional factor activation. Activity was normalized with transcription factor IID, and expressed as the folds of control. **b** Mouse BM-derived macrophages were transduced with lentivirus that expresses CMV promoter-driven renilla luciferase (CMV-rLuc), hypoxia response element-driven firefly luciferase (HRE-fLuc), and mutated HRE-fLuc (muHRE-fLuc), followed by treatment with CSF-1 and IL-6 for 2 days. Reporter activity radio of fLuc versus rLuc was determined by bioluminescence. Results were expressed as the percentage of muHRE (*n* = 3, mean ± SEM). *P* value was determined by Student’s *t* test. **c**–**e** Mouse BM-derived macrophages were transduced with lentivirus that expresses shRNAs targeting control scrambled sequence, HIF-1α (#49 and #52) and HIF-2α (#5 and #7), followed by treatment with CSF-1 and IL-6 for 10 days. **c** Cells were lysed and subjected to immunoblot analysis. **d** Arginase-1 mRNA was analyzed by real-time RT-PCR. Shown are quantified data (normalized with GAPDH expression, *n* = 3, mean ± SEM). **e** Cells were stained with anti-CD11b, anti-CD86, and anti-CD206 antibodies, followed by flow cytometry analysis (*n* = 3, mean ± SEM)

To explore the mechanisms by which CSF-1 and IL-6 induce arginase-1 expression and macrophage alternative activation, we performed a multiplex screening assay for the DNA-binding activity of 96 transcriptional factors in the treated macrophages. Unexpectedly, IL-6 treatment alone decreased the activities of most transcriptional factors (Fig. 5a). However, co-treatment of CSF-1 and IL-6 activated multiple transcriptional factors, including AP1, HIF (hypoxia-inducible factor), KLF4, NF-κB, and PPAR (peroxisome proliferator-activated receptor) that have previously been shown critical for macrophage alternative activation^24, 27^. In contrast, CSF-1 treatment alone slightly activated HIF, KLF4, and PPAR, while inactivated AP1 and NF-κB. Notably, CSF-1 and IL-6 co-treatment remarkably increased the DNA-binding activity of HIF and PPAR, as indicated by about 70-fold and 80-fold increase in their activity, respectively.

We first tested the role of HIFs in macrophage alternative activation. HIFs are heterodimers comprising one of three major oxygen labile HIF-α subunits (HIF-1α, HIF-2α, or HIF-3α), and a constitutive HIF-1β subunit. HIFs are crucial mediators of hypoxic response, which transcribe a large number of genes that promote angiogenesis, anaerobic metabolism, and resistance to apoptosis, and therefore crucial for cancer development and progression^28–30^. We verified the CSF-1- and IL-6-induced HIF transcriptional activation by HIF-responsive element (HRE)-driven luciferase expression analysis (Fig. 5b). HIF-1α and HIF-2α expression is known to be regulated by hypoxia-induced protein stability, but could also be induced by oxidative stress, growth factors, and succinate under normoxia^31–35^. Here we showed that CSF-1 and IL-6 remarkably increased protein expression of HIF-2α, but not HIF-1α, under normoxia in mouse macrophages (Fig. 5c) and human monocytes (Supplementary Fig. 9). Furthermore, the co-treatment stimulated expression and nuclei translocation of HIF-2α, but not HIF-1α (Supplementary Fig. 10), supporting the increased HIF transcriptional activity and the distinct HIF-2α expression induced by CSF-1 and IL-6.

A recent study suggests a critical role of HIF-2α in macrophage alternative activation^36^. Consistently, our data show that short hairpin RNA (shRNA)-mediated HIF-2α knockdown almost completely blocked CSF-1-induced and IL-6-induced arginase-1 protein and mRNA expression (Fig. 5c, d) and macrophage alternative activation (Fig. 5e). In addition, shRNA-mediated knockdown of IL-6 receptor-α and IL-6 neutralization significantly inhibited EC-CM-induced PPARγ, HIF-2α, and arginase-1 expression, suggesting a critical role of IL-6 for activation of the HIF-2α/arginase-1 axis (Supplementary Fig. 11). Together, these data identify a critical role of HIF-2α in CSF-1-induced and IL-6-induced macrophage alternative activation.

### PPARγ is required for HIF-2α and arginase-1 expression

**Fig. 6:**
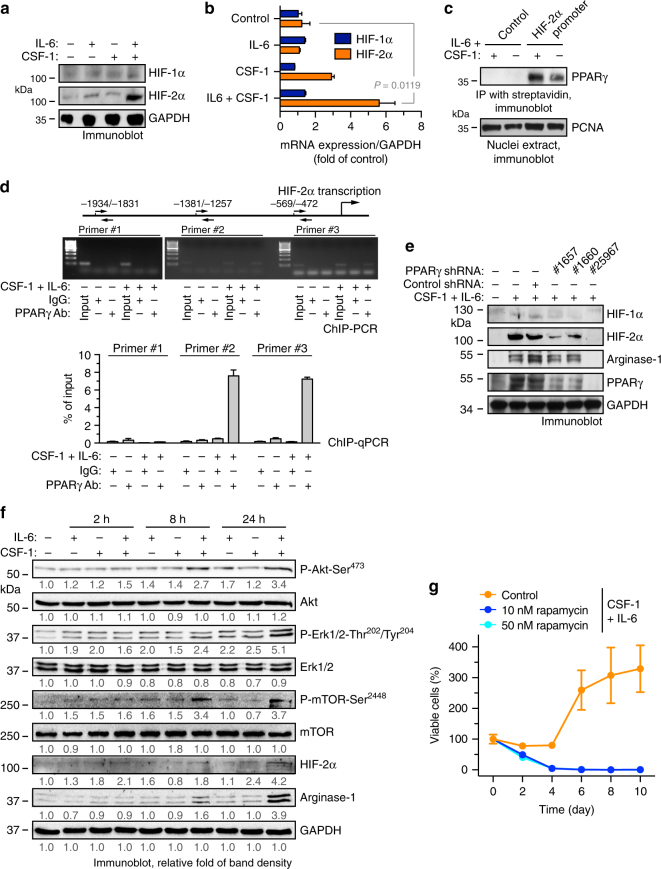
PPARγ induces HIF-2α and arginase-1 expression in macrophages. **a**,** b** Mouse BM-derived macrophages were treated with or without CSF-1 and IL-6 for 5 days. **a** Cell lysates were immunoblotted. **b** mRNA was extracted and subjected to quantitative RT-PCR analysis. Results were normalized with GAPDH level and expressed as folds of control (*n* = 3, mean ± SEM). *P* value was determined by Student’s *t* test. **c**, **d** Mouse BM-derived macrophages were treated with or without CSF-1 and IL-6 for 3 days. **c** Nuclei protein was incubated with biotin-labeled synthetic DNAs that encode control scrambled or HIF-2α promoter sequence, followed by immunoprecipitation with streptavidin-conjugated beads. Precipitants and nuclei protein were immunoblotted. **d** Nuclei extracts were subjected to chromatin immunoprecipitation (ChIP) analysis. Immunoprecipitants with control IgG or anti-PPARγ antibody were analyzed by PCR and electrophoresis (upper) or by quantitative PCR (bottom, *n* = 3, mean ± SD). **e** Mouse BM-derived macrophages were transduced with lentivirus that expresses shRNAs targeting control scrambled sequence and PPARγ (#1657, #1660, and #25967), followed by treatment with CSF-1 and IL-6 for 10 days. Cells were lysed and subjected to immunoblot analysis. **f** Mouse BM-derived macrophages were treated with or without CSF-1 and IL-6. At different time points post-treatment, cells were lyzed and subjected to immunoblot analysis. Band density was quantified. **g** Mouse BM cells were pretreated with or without rapamycin, followed by incubation with CSF-1 and IL-6. Cell viability was determined (*n* = 3 mice, mean ± SEM)

Interestingly, co-treatment with IL-6 and CSF-1, but not either one alone, remarkably enhanced protein and mRNA expression of HIF-2α (Fig. 6a, b), suggesting that the co-treatment upregulates HIF-2α expression through modulating HIF-2α transcription. We analyzed the promoter sequence of HIF-2α (EPAS1) and predicted that the transcriptional factors including SP1, PPARγ, GATA-3, and HOXA9 may possibly bind to the region based on motif recognition pattern. We explored the regulatory mechanism for HIF-2α transcription with a focus on PPARγ, considering a robust activation of PPAR induced by the co-treatment (Fig. 5a) and an established role of PPARγ in macrophage alternative activation^37, 38^. Our data indicated that the co-treatment stimulated HIF-2α promoter interaction with PPARγ, as shown by an immunoprecipitation analysis using synthetic biotin-labeled HIF-2α promoter DNA (Fig. 6c and Supplementary Fig. 12). Moreover, chromatin immunoprecipitation (ChIP) analysis validated that IL-6 and CSF-1 co-treatment induced HIF-2α binding to PPARγ promoter in macrophages (Fig. 6d). Furthermore, shRNA-mediated knockdown of PPARγ inhibited IL-6 and CSF-1 co-treatment-induced expression of arginase-1 and HIF-2α (Fig. 6e), suggesting that PPARγ regulates arginase-1 and HIF-2α expression in macrophages.

IL-6 induces inflammatory responses via JAK/STAT signaling and promotes cell survival and growth via MAPK-mediated and Akt-mediated pathway, respectively. CSF-1 is also known to be able to activate these survival and growth signaling pathways and also induces PPARγ activation. We investigated these signaling events with time resolution in macrophages co-treated with IL-6 and CSF-1. Co-treatment with CSF-1 increased IL-6-induced Akt1, Erk, and mTOR signaling activation (Fig. 6f). Furthermore, treatment with LY294002 and rapamycin, pharmacological inhibitors of PI3K/Akt and mTOR, respectively, or shRNA-mediated PPARγ knockdown, inhibited IL-6-induced and CSF-1-induced macrophage proliferation (Fig. 5g and Supplementary Fig. 13), suggesting that Akt/mTOR/PPARγ is required for IL-6-induced and CSF-1-induced macrophage growth. Together, we propose a model for co-treatment-induced signaling mechanism for macrophage alternative activation: co-treatment induces arginase-1 expression through PPARγ and HIF-2α, leading to macrophage alternative activation, and simultaneously it activates Akt1/mTOR to promote survival and growth of the alternatively activated macrophages in the tumor microenvironment.

### ECs are one of major sources of IL-6 expression in GBM

**Fig. 7:**
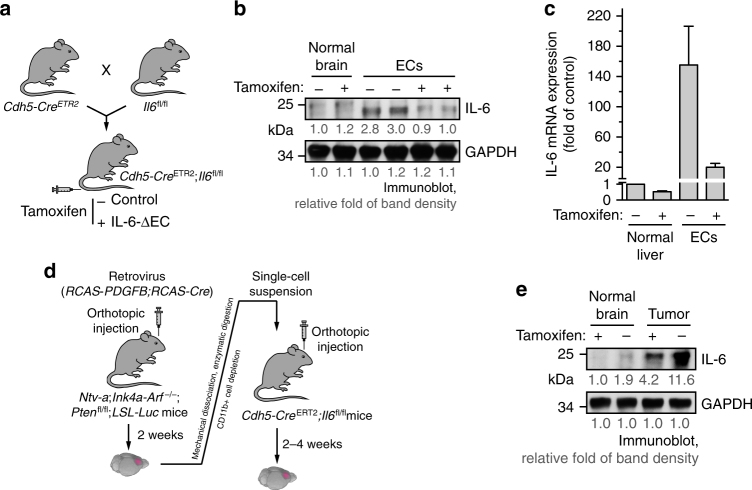
ECs are a major source for IL-6 expression in GBM. **a**–**c** *Cdh5*-*Cre*^ERT2^;*Il6*^fl/fl^ mice were generated by crossing *Cdh5*-*Cre*^ERT2^ mice with *Il6*^fl/fl^ mice. Mice (2 weeks old) were injected with tamoxifen for consecutive 5 days to induce EC-specific IL-6 knockout. **a** Schematic approach. **b**, **c** ECs were isolated from mouse aortas. **b** Brain tissue and ECs were subjected to immunoblot analysis. Band density was quantified. **c** mRNA was extracted and subjected to quantitative RT-PCR analysis. Results were normalized with GAPDH level and expressed as folds of control (*n* = 3, mean ± SEM). **d**, **e** The primary GBM in *Ntv-a*;*Ink4a-Arf*^−/−^;*Pten*^−/−^;*LSL-Luc* donor mice was induced by RCAS-mediated somatic gene transfer. Recipient mice were *Cdh5*-*Cre*^ERT2^;*Il6*^fl/fl^ mice. **d** Schematic approach. **e** Normal brain and tumor tissues were homogenized. Tissue lysates were immunoblotted. Band density was quantified

Previous immunohistochemial studies with human GBM specimens suggest tumor-associated ECs and inflammatory cells as the major sources for IL-6 expression in the tumor microenvironment^39^. Consistently, we showed that GBM-associated ECs expressed IL-6 at a relatively higher level, compared with U251 glioma cells, T4123 glioma stem cells, and human PBMC monocytes (Supplementary Fig. 14). To rigorously investigate the in vivo role of ECs in IL-6 expression as well as IL-6’s functions in GBM progression, we generated a conditional IL-6 knockout *Cdh5*-*Cre*^ERT2^;*Il6*^fl/fl^ mouse line, in which IL-6 knockout was induced by tamoxifen-inducible Cre^ERT2^ expression under EC-specific Cdh5 promoter (Fig. 7a). The efficient and selective knockout was validated by immunoblot and quantitative reverse transcription-PCR (RT-PCR) in aortic and brain ECs (Fig. 7b, c and Supplementary Fig. 15). We took advantage of the orthotopic, genetic murine GBM model with a native microenvironment, induced by *RCAS*/*N-tva*-mediated somatic PDGF gene transfer in *Ink4a-Arf*^−/−^;*Pten*^−/−^ neural stem/progenitor cells (Fig. 7d), followed by tumor transplantation into the new generated *Cdh5*-*Cre*^ERT2^;*Il6*^fl/fl^ mice. Notably, the genetic GBM mouse model is immunocompetent, which recapitulates the major features of human GBM including pseudopalisading necrosis and microvascular proliferation^35, 40^. Immunoblot analysis of these tumors reveals that IL-6 deletion in ECs significantly reduced IL-6 expression in whole tumor lysates (Fig. 7e), confirming that ECs are one of major sources of IL-6 expression in GBM microenvironment.

### Endothelial IL-6 is critical for GBM growth and progression

**Fig. 8:**
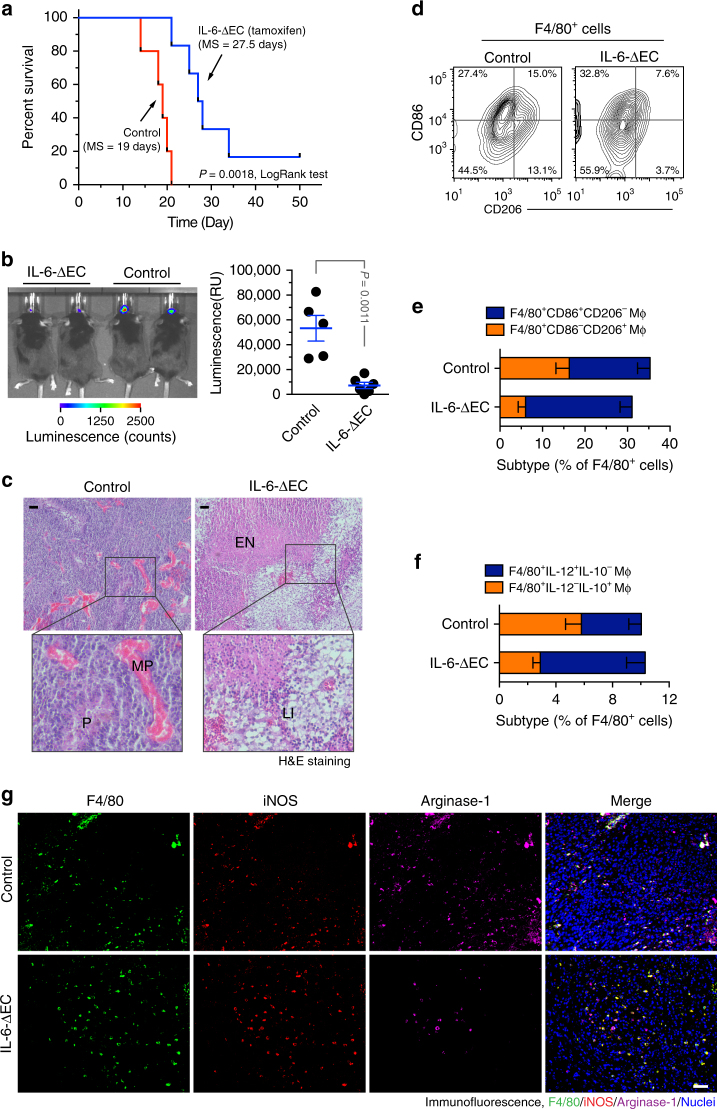
Endothelial IL-6 is critical for macrophage alternative activation and GBM growth and progression. The genetically engineered GBM model was induced in *Ntv-a*;*Ink4a-Arf*^−/−^;*Pten*^−/−^;*LSL-Luc* donor mice, followed by orthotopic tumor implantation into *Cdh5*-*Cre*^ERT2^;*Il6*^fl/fl^ mice that were treated with (IL-6-ΔEC) or without (Control) tamoxifen. **a** Animal survival was monitored for 50 days post-injection (*n* = 5–6 mice, one representative result from three independent experiments). *P* values were determined by log-rank (Mantel–Cox) tests. MS, median survival. **b** Tumor growth was analyzed by bioluminescence. Left, representative images. Right, quantitative analysis of integrated luminescence in tumors at day 12 (mean ± SEM, *n* = 5–6, one representative result from three independent experiments). *P* value was determined by Student’s *t* test. **c** Tumor sections were stained with hematoxylin and eosin (H&E). Representative images are shown (*n* = 10 mice). P pseudopalisades, MP microvascular proliferation, EN extensive necrosis, LI leukocyte infiltration. Bar represents 100 μm. Zoom-in factor: 3. **e**, **f** Tumors were excised. Single-cell suspensions were prepared and subjected to flow cytometry analysis. **d**, **e** Single-cell suspensions were probed with anti-F4/80, anti-CD86, and anti-CD206 antibodies. CD206 and CD86 expression were analyzed in sorted F4/80^+^ cells. **d** Representative sorting. **e** Quantified results (mean ± SEM, *n* = 10–14 mice). **f** Single-cell suspensions were probed with anti-F4/80, anti-IL-10, and anti-IL-12 antibodies. IL-10 and IL-12 expression was analyzed in sorted F4/80^+^ cells. Show are quantified results (mean ± SEM, *n* = 8–13 mice). **g** Tumor sections were stained and analyzed by immunofluorescence. Tumor sections were probed with anti-iNOS, anti-arginase-1, anti-F4/80 antibodies (*n* = 10 mice). Bar represents 100 μm

We finally investigated the in vivo role of endothelial IL-6 in GBM progression. Our data show that IL-6 deletion in ECs significantly improved animal survival in the mice bearing the genetically induced GBM tumors, leading to an increase in overall survival time by 45% (19 versus 27.5 days, in control and tamoxifen-treated *Chd5-Cre*^ERT2^;*Il6*^fl/fl^ mice, Fig. 8a). Notably, about 20% of mice survived through the experimental process, showing no detectable tumors when euthanized at day 50. Consistent with the critical role of endothelial IL-6 in mouse survival, EC-specific knockout of IL-6 significantly reduced tumor growth, as indicated by a 70% decrease in average tumor volume (at day 12 after tumor implantation, Fig. 8b). Furthermore, IL-6 knockout eliminated pseudopalisades (P) and microvascular proliferation (MP) in the tumors (Fig. 7c), suggesting a critical role of EC-derived IL-6 in GBM progression.

Strikingly, IL-6 knockout robustly induced extensive necrosis and leukocyte infiltration (Fig. 8c), possibly due to its negative effects on macrophage-mediated tumor immunity. Consistently, our data showed more neutrophils and CD3^+^ T cells infiltrated in the tumors (Supplementary Fig. 16). Flow cytometry analysis of tumor-associated F4/80^+^ macrophage cells show that indicated that IL-6 knockout robustly decreased CD86^−^CD206^+^ macrophage population and slightly increased CD86^+^CD206^−^ macrophage population (Fig. 8d, e). Moreover, IL-6 deletion in ECs increased the expression of proinflammatory IL-12 and reduced the expression of anti-inflammatory IL-10 (Fig. 8f). Futhermore, IL-6 deletion did not affect iNOS expression, but remarkedly inhibited arginase-1 expression in tumor-associated F4/80^+^ macrophages (Fig. 8g), supporting the important role of endothelial IL-6 in macrophage alternative activation. Together, these data suggest that endothelial cell-derived IL-6 is critical for macrophage alternative polarization and GBM progression.

## Discussion

**Fig. 9:**
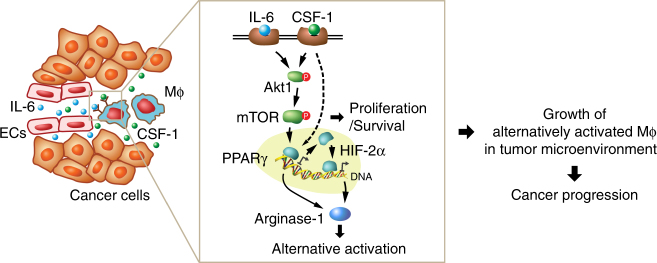
A schematic model. In glioma microenvironment, endothelial cell-derived IL-6 and microenvironmental CSF-1 synergistically activate downstream Akt1/mTOR pathway and induces transcriptional activation of PPARγ in macrophages (Mϕ), in turn leading to HIF-2α-mediated arginase-1 expression, and inducing macrophage alternative polarization. The activation of mTOR also induces cell proliferation, contributing to cell survival and growth of alternatively activated macrophages, eventually leading to glioma progression

Our studies identify a vascular niche that drives macrophage alternative polarization and GBM progression. Namely, tumor-associated ECs express and release IL-6, jointing with CSF-1 in the microenvironment, induces HIF-2α-dependent arginase-1 expression through activation of PPAR-γ, leading to macrophage alternative polarization and GBM progression. IL-6 and CSF-1 also induce mTOR activation, resulting in cell survival and growth of alternatively activated macrophages in the tumor microenvironment (Fig. 9).

Macrophages are a crucial player in tumor–host immune interaction and cancer development and progression. Functional polarization, for example, M0 (naïve status), M1 (classically activated), or M2 (alternatively activated), of macrophages represents a key mechanism that controls their functions, switching roles between tumor suppressing and promoting^4, 5, 41^. Although it has been well established that most of glioma-associated macrophages acquire alternative activation to execute tumor-promoting and tumor-immunosuppressive functions^23, 42^, recent ex vivo studies based on transcriptome analysis of cytokine and cell marker expression in isolated myeloid cells suggest that glioma-associated macrophages exhibit a M0 polarization profile^43^, suggesting that classical or alternative activation of macrophages depends on the stimulus from tumor microenvironment and also are possibly not driven by cell plasticity-mediated permanent cell fate transition.

Vascular ECs are a major component of tumor microenvironment. In addition to their classical functions for delivering oxygen and nutrients to support tumor growth, tumor-associated ECs act as a niche that fuels tumor growth, progression, and metastasis by producing paracrine factors, that is, angiocrines, to tumor microenvironment^40, 44–52^. For example, it is well known that perivascular niche is critical for stemness maintenance and self-renewal in cancer stem cells^44, 47, 50^. The role of vascular niche in tumor immunity regulation, nevertheless, remains unidentified. Here our study reveals that EC-derived IL-6 promotes macrophage alternative activation and GBM progression in vivo, therefore identifying a vascular niche that controls macrophage functions in cancer. Supportive to this concept, a recent study shows that in vitro co-culture with ECs induces differentiation of hematopoietic progenitor and stimulates macrophage alternative activation^53^.

Previous work shows that genetic IL-6 ablation blocks tumor formation of spontaneous mouse glioma driven by glial fibrillary acidic protein-mediated expression of viral *Src* oncogene^54^. Our work reveals that EC-derived IL-6 is critical for tumor growth and progression in a genetically induced GBM mouse model. Importantly, analyses of TCGA and Rembrandt databases show that elevated IL-6 expression correlates with poor overall survival in glioma and GBM patients (Supplementary Fig. 17). Consistently, IL-6 blockade treatment significantly improved animal survival time by 35% in our genetically engineered GBM model (17 versus 23 days, in control and anti-IL-6 antibody-treated mice, Supplementary Fig. 18). Thus, IL-6 blockade may serve as an efficient strategy for therapeutic intervention of GBM. In addition, IL-6-deficient mice develop normally^55^. As such, its EC-preferential induction in tumor microenvironment, but dispensability in development, suggests that IL-6 may present a highly selective and non-noxious therapeutic target for cancer treatment.

Alternative macrophage activation is known to be driven by multiple cytokines including IL-4 and IL-13^6, 22^. Here we identify IL-6 as a major driver for alternative macrophage activation in glioma microenvironment. IL-6 has classically been recognized as a proinflammatory cytokine^56, 57^. As such, IL-6 receptor blockade has been served as an important therapeutic strategy for the treatment of inflammatory diseases including rheumatoid arthritis^58^. Interestingly, IL-6 regulates T cell proliferation and survival^56, 59^, induces IL-4 production by CD4^+^ T cells^60^, and promotes T cell Th17 differentiation^61^. A recent study reports that IL-6 induces IL-4 receptor expression in macrophages, leading to their alternative activation in diabetic inflammation^26^, suggesting an unexpected role for IL-6 in macrophage-mediated anti-inflammatory responses. Likewise, our data for the first time identify IL-6 as a critical instigator of macrophage alternative activation in cancer. We show that IL-6 and CSF-1 induce robust arginase-1 expression that primes macrophages to alternative activation. In addition, previous studies reveal that IL-6 enhances expression of IL-4 and IL-13, two stimulators for macrophage alternative activation, likely secreted by T cells^60^, which may further promote macrophage alternative activation in the tumor microenvironment.

Macrophage polarization is subjected to transcriptional regulation driven by cues in the tissue microenvironment^27^. Activation of multiple transcriptional factors, mainly induced by IL-4 and IL-13, drives macrophage alternative activation, which includes AP1, HIF-2α, KLF4, PPARγ, and STAT-6^27, 37, 38, 62,-64^. The mechanisms that control macrophage activation in cancer, particularly induced by IL-6, remain largely unknown. Here we reveal that IL-6 and CSF-1 induce PPARγ-dependent HIF-2α transcription, leading to arginase-1 expression and macrophage alternative activation in GBM. Interestingly, a recent work that has screened 270 transcriptional factors shows that PPARγ coactivator/estrogen-related receptor induces HIF-2α expression in neuroblastoma, independent of canonical hypoxia-mediated regulation of protein stability, but through transcription regulation^65^, supporting our observation of HIF-2α mRNA expression induced by PPARγ in GBM.

In summary, our study identifies a vascular niche for the regulation of tumor immunity and reveals an IL-6-mediated mechanism controlling macrophage alternative activation and GBM progression. Specifically, IL-6 and CSF-1 induce PPARγ-dependent HIF-2α transcription, leading to arginase-1 expression and macrophage alternative polarization in GBM. Thus, targeting IL-6 may offer exciting therapeutic opportunities to reactivate macrophage-mediated tumor immunity, which may block tumor progression and treatment resistance.

## Methods

### Patient and tumor endothelial cell sorting

All patients received surgery at the Department of Neurosurgery of the University of Pennsylvania and were enrolled in a single institution tissue banking protocol that was approved by the University of Pennsylvania Human Studies Committee. Consent was obtained from all patients. Tumor-associated ECs were isolated as previously described^66^. In brief, tumor-derived single-cell suspensions were prepared by the tissue bank. Red cells were removed with ACK lysis buffer (Life Technologies). Cell suspension was subjected to magnetic activating cell sorting (MACS) with anti-CD31 antibody-conjugated magnetic beads (Miltenyi Biotech, 130-091-935). Sorted ECs were verified by Dil-Ac-LDL (Bioquote) absorption, and over 99% of cells were Dil-Ac-LDL-positive.

### Cell culture

Human and mouse brain microvascular ECs (ScienCell and PromoCell) were maintained in Endothelial Cell Medium (ECM, ScienCell) at 37 °C in a humidified air atmosphere with 5% CO_2_. All cells were used between passages 2 and 5. GL26 mouse glioma cells were kindly provided by Chunsheng Li (University of Pennsylvania) and cultured in Dulbecco's modified Eagle's medium (DMEM) medium (Gibco) supplemented with 5% fetal bovine serum (FBS). Chicken DF-1 fibroblasts (ATCC) were maintained in DMEM medium (ATCC, 30-2002) containing 5% FBS at 39 °C in a humidified air atmosphere with 5% CO_2_. T4123 glioma stem cells were kindly provided by Jeremy Rich (Cleveland Clinic) and then cultured in serum-free Neurobasal-A medium (Gibco), supplemented with B-27 Supplement Minus Vitamin A (Gibco), GlutaMax (Gibco), sodium pyruvate (Gibco), fibroblastic growth factor (5 ng/ml, R&D Systems), and EGF (20 ng/ml, R&D Systems). All cancer cell lines were checked and showed no mycoplasma contamination.

### Mice

Wild-type (WT) mice on the C57BL/6J background were obtained from Jackson Lab. *Cdh5-Cre*^ERT2^ mice were generated by Ralf Adams (Max Planck) and kindly provided by Nancy Speck (University of Pennsylvania) and Bisen Ding (Cornell)^67^. *Il6*^fl/fl^ mice have been described previously^68, 69^. *Cdh5-Cre*^ERT2^;*Il6*^fl/fl^ mice were generated by crossing *Il6*^fl/fl^ mice with *Cdh5-Cre*^ERT2^ mice. Mice were genotyped with primers including IL-6 FP: 5′-CCCACCAAGAACGATAGTCA-3′, and IL-6 RP: 5′-GGTATCCTCTGTGAAGTCCTC-3′. *Cdh5-Cre*^ERT2^;*Il6*^fl/fl^ and *Il6*^fl/fl^ mice (2 weeks old) were intraperitoneally injected with 0.1 ml of 5 mg/ml tamoxifen daily for consecutive 5 days. *Rosa-LSL-tdTomato*;*Tie2-Cre* mice were generated by crossing *Rosa-LSL-tdTomato* mice (Jackson Lab) with *Tie2-Cre* mice (Jackson Lab). All animal studies were reviewed and approved by the Institutional Animal Care and Use Committees (IACUC) at the University of Pennsylvania. All animals were housed in the Association for the Assessment and Accreditation of Laboratory Animal Care-accredited animal facility of the University of Pennsylvania.

### Isolation and culture of mouse ECs

Mouse aortic ECs were isolated from *Cdh5-Cre*^ERT2^;*Il6*^fl/fl^ mice as previously described^35^. In brief, thoracic aorta was isolated from 3-week-old mice and cut into pieces. Aortic rings were embedded in phosphate-buffered saline (PBS)-prewashed Matrigel and then incubated in DMEM/F-12 medium containing 5% FBS for 5 days. After rinsing with PBS, the rings were removed and the remaining cells were incubated with 2 U/ml Dispase I (Gibco, 17105-041) for 20 min at 37 °C. After centrifugation at 500 × *g* for 10 min, the cell pellets were washed with PBS. ECs were also isolated from mouse brain. Single-cell suspension was prepared and subjected to MACS with anti-CD31 antibody-conjugated magnetic beads (Miltenyi Biotech, 130-091-935). Cells were cultured in DMEM/F-12 medium supplemented with 25 μg/ml EC growth supplement (Sigma) and 5% FBS. All cells were used between passages 2 and 4.

### Genetic GBM mouse model

GBM was induced in mice as previously described^66^. In brief, chicken DF-1 fibroblasts (ATCC) were transfected with RCAS-PDGF-B and RCAS-Cre plasmids to produce retrovirus, and orthotopically injected into *Ntv-a*;*Ink4a-Arf*^−/−^;*Pten*^fl/fl^*;LSL-luc* mice to induce GBM through RCAS/n-tva-mediated gene transfer. Tumors were isolated and subjected to mechanical dissociation with a gentleMACS Dissociator (Miltenyi Biotech) and enzymatic digestion with collagenase II and dispase II to obtain single-cell suspensions. About 8 weeks old *Cdh5-Cre*^ETR2^;*Il6*^fl/fl^ and *Il6*^fl/fl^ mice, half male and half female, were orthotopically and stereotactically injected with 10^5^ GBM tumor cells that express luciferase. Tumor growth was monitored by whole-body bioluminescence using an IVIS 200 Spectrum Imaging System after retro-orbital injection of luciferin (150 mg/kg, GoldBio). Survival was monitored for 50 days. For antibody treatment experiment, tumor-bearing mice were intraperitoneally treated with anti-CSF-1 (BioXcell, BE0204, 1 mg per mouse x 4) or anti-IL-6 (BioXcell, BE0046, 200 μg per mouse x 4) antibody or control rat immunoglobulin G (IgG) (BioXcell, BE0090, 200 μg per mouse x 4). Mice were euthanized when exhibiting severe GBM symptoms including domehead, hemiparesis, or more than 20% of body weight loss. Mice were randomized to receive treatment, and the investigators were not blinded.

### GL26 glioma mouse model

GL26 glioma was induced in mice as previously described^66^. In brief, about 8 weeks old *Rosa-LSL-tdTomato*;*Tie2-Cre* and WT mice were orthotopically and stereotactically injected with 10^5^ GL26 mouse glioma cells that express luciferase. Tumor growth was monitored by whole-body bioluminescence using an IVIS 200 Spectrum Imaging System after retro-orbital injection of luciferin (150 mg/kg, GoldBio).

### Preparation of glioma-CM

Mouse GL26 glioma cells or Human U251, U87, and IN528 glioma cells were cultured with DMEM medium supplemented with 5% FBS. Cells at about 90% confluence were exposed to hypoxia (1% O_2_) in a humidified air atmosphere at 37 °C for 24 h. Culture medium were centrifuged at 5,000 × *g* for 30 min to remove cellular debris and then the supernatant was collected.

### Preparation of EC-CM

Human normal brain microvascular ECs (ScienCell and PromoCell) and GBM patients' tumor-derived ECs (#5377, #5441, #5391, #5465) were maintained with ECM (ScienCell). When reaching about 70% confluence, cells were cultured at 37 °C for 24 h. Culture medium were centrifuged at 5,000 × *g* for 30 min to remove cellular debris and then the supernatant was collected.

### BMDM isolation and treatment

Freshly isolated femur and tibia from WT C57BL/6 mice were flushed with RPMI-1640 medium (Life Technologies). Cells were harvested and passed through a 40-μm strainer. Red cells were removed with ACK lysis buffer. BM cells were cultured in RPMI-1640 medium supplemented with 5% FBS (Life Technologies). Cells were incubated with 10 ng/ml human CSF-1 (BioLegend, 574808) for 7 days to induce macrophage differentiation. Cells were treated with 100 ng/ml mouse IL-6 (BioLegend, 575708) and CSF-1 for different times.

### Human peripheral blood-derived monocytes and treatment

Primary human monocytes, purchased from the Human Immunology Core at the University of Pennsylvania, were isolated from healthy volunteer donors following leukapheresis by negative selection. All specimens were collected under a University Institutional Review Board-approved protocol, and written informed consent was obtained from each donor. Isolated cells were incubated in RPMI-1640 medium supplemented with 5% heat-inactivated FBS. Cells were treated with 10 ng/ml human CSF-1, 100 ng/ml human IL-6 (BioLegend, 570808), and normal human ECs or patient tumor EC-CM.

### Co-culture of BM cells and ECs and treatment

Mouse BM cells were co-cultured with mouse brain microvascular ECs, CCL5 (PeproTech, 250-07), or CXCL5 (PeproTech, 300-22) under normoxia or hypoxia (1% O_2_). ECs were seeded in 6-well plates (5 × 10^3^/well) and then cultured overnight with DMEM medium containing 5% FBS, followed by treatment with glioma-CM as described above. ECs were washed with PBS and co-cultured with freshly prepared BMDMs (10^4^ cells per well) for 5 days in RPMI-1640 media supplemented with 5% FBS. Cells were treated with specific neutralizing antibodies against CSF-1 (1 μg/ml, R&D Systems, AF416) and IL-6 (1 μg/ml, BioLegend, 504505), or control IgG (Sigma). Cells were fixed and subjected to immunofluorescence analysis. Single-cell suspension was prepared by using Versene solution (0.02% EDTA, Thermo) and then subjected to flow cytometry analysis.

### shRNA and siRNA treatment

The 293T cells (ATCC) were co-transfected with packaging vectors (System Biosciences) and lentiviral expression vectors that encode shRNA targeting HIF-1α and HIF-2α (kindly provided by Celeste Simon as previously published^70^), and PPARγ (Sigma, TRCN0000001657, TRCN0000001660, and TRCN0000025967) for 8 h. For IL-6R-α knockdown, the oligonucleotides (#1: sense, 5′-TACCGACCTGTATGGTCAAATTCAAGAGATTTGACCATACAGGTCGGTTTTTTTC-3′, and antisense, 5′-TCGAGAAAAAAACCGACCTGTATGGTCAAATCTCTTGAATTTGACCATACAGGTCGGTA-3′; #2: sense: 5′-TATCAGTACGAAAGTTCTACTTCAAGAGAGTAGAACTTTCGTACTGATTTTTTTC-3′, antisense, 5′-TCGAGAAAAAAATCAGTACGAAAGTTCTACTCTCTTGAAGTAGAACTTTCGTACTGATA-3′; #3: sense, 5′-TCAATACCGTAAACCACAGCTTCAAGAGAGCTGTGGTTTACGGTATTGTTTTTTC-3′, antisense, 5′-TCGAGAAAAAACAATACCGTAAACCACAGCTCTCTTGAAGCTGTGGTTTACGGTATTGA-3′) that encode shRNAs were synthesized and cloned into the pSicoR lentivirus expression vector, followed by transfection with 293T cells. After change with fresh medium and incubation for 48 h, medium supernatants containing lentivirus were collected. Freshly separated BMDMs were infected with lentivirus in the presence of 8 μg/ml polybrene (Millipore). Stably shRNA-expressing cells were selected with 1.8 μg/ml puromycin and maintained in the culture medium with 0.5 μg/ml puromycin. Cells were transfected with control or IL-6 siRNA (Ambion, 4390771) by using Lipofectamine 2000 (Invitrogen).

### Dual luciferase reporter assay

WT or mutated HRE with firefly luciferase reporter in pGL2 (kindly provided by Celeste Simon) were subcloned into lentivirus expression vector pCDH (System Biosciences). The 293T cells (ATCC) were co-transfected with packaging vectors (System Biosciences) and lentiviral expression vectors that express HRE-luciferase or control CMV-renilla luciferase. Freshly prepared BMDMs were treated and transduced with lentivirus, followed by measurement of luciferase activity using a Dual Luciferase Assay System (Promega). Each measured firefly luciferase activity was normalized by the renilla luciferase activity in the same well.

### Transcription factor activation profiling array

BMDMs were treated with different cytokines. Nuclear proteins were isolated with a nuclear extraction kit (Signosis, SK-0001), and analyzed by using a 96-well plate transcription factor (TF) activation array (Signosis, FA1002) according to the manufacturer’s instruction. The activity of each transcriptional factor was normalized as the fold of TFIID’s activity.

### Chromatin immunoprecipitation

ChIP assays were performed using a Magna ChIP kit (Millipore, MAGNA0001) according to the manufacturer’s instructions. Mouse BM cells (10^7^ cells) cultured in 15-cm dishes were treated with CSF-1 and IL-6. Cells were crosslinked with 1% formaldehyde for 10 min at room temperature, followed by glycine incubation for 5 min. The cells were sonicated three times, each for 16 s, using a W-385 sonicater (Heat Systems Ultrasonics). Immunoprecipitants with chromatin were prepared by using 20 μg anti-PPARγ (Santa Cruz, sc-7196) or anti-normal rabbit IgG (Santa Cruz, sc-2027) with cell lysates from 10^6^ cells in 0.5 ml reaction buffer. The primers pairs for HIF-2α promoter used in the ChIP-PCR and ChIP-qRT-PCR are listed as followings: Primer #1: FP, 5′-TACAGCTCAAATCCAGCAGAAGC-3′, RP, 5′-GAGGGAGGGAAAGACCAGACAATAA-3′; Primer #2: FP, 5′-GCTTTCTCCGCAATTCACAACTATG-3′, RP, 5′-TCTTAGACACTTTCCCATTTCCTACTT-3′; Primer #3: FP, 5′-CGCCATTACTCAGTCCTGCGCTAA-3′, RP, 5′-TTCCGCAGAACTGCGACTTGTTT-3′.

### Cytokine array

Human brain microvascular ECs were treated with glioma-CM or control medium for 24 h at 37 °C. After washing twice with PBS, cells were incubated with DMEM/F-12 medium supplemented with 2% bovine serum albumin (Sigma) for 12 h. Cell lysates were subjected to multiplex cytokine analysis using a Human XL Cytokine Array (Ary022, R&D Systems) according to the manufacturer's instructions.

### Quantitative RT-PCR analysis

Total RNA was isolated using TRIzol reagent (Invitrogen) from treated mouse BMDMs, mouse aortic ECs, and mouse liver tissue. RNA was reversely transcribed using SuperScript III First-Strand Synthesis SuperMix (Life Technologies), and subjected to real-time PCR analysis using TaqMan^®^ Fast Universal PCR Master Mix (Life Technologies) and primers: HIF-1α (Applied (Biosystems, Mm00468869_m1), HIF-2α (EPAS1, Applied Biosystems, Mm00438717_m1), IL-6 (Applied Biosystems, Mm00446190_m1), and arginase-1 (FP: 5′-CTCCAAGCCAAAGTCCTTAGAG-3′; RP: 5′-GCATCCACCCAAATGACACAT-3′). Quantitative RT-PCR was performed in a 20-μl reaction volume using Fast SYBR Green Master Mix (Applied Biosystems) with 18S rRNA or glyceraldehyde 3-phosphate dehydrogenase (GAPDH) used as endogenous controls.

### Immunofluorescence and histology

Human surgical specimens from subjects with glioma or normal brain tissues (from US Biomax, BioChain, and from patients treated in Department of Neurosurgery, Sun Yat-sen University Cancer Center, China. Patient specimens were collected under a University Institutional Review Board-approved protocol, and written informed consent was obtained from each patient), or mouse tumors tissues were used. Paraffin sections were de-paraffinized and rehydrated, and subjected to antigen retrieval in Target Retrieve Solution (Dako, S1699) at 95 °C for 20 min. Sections were blocked with 5% horse serum for 1 h at room temperature. Human samples were incubated with anti-CD31 (1:100, Cell Signaling, 3528), anti-CD68 (1:100, Cell Signaling, 76437), anti-CD86 (1:100, BD Pharmingen, 555656), anti-CD163 (1:100, Serotec/Bio-Rad, MCA1853T), anti-CD206 (1:100, Santa Cruz, SC376108), anti-arginase-1 (1:100, Santa Cruz, SC20150), or anti-iNOS (Abcam, ab3523) antibody overnight at 4 °C. For mouse tissues, sections were incubated with anti-CD31 (1:100, Dianova, DIA310), anti-IL-6 (1:100, Dako, A0082; Novus, NB600-1131), anti-CSF-1 (1:100, Millipore, AB5320), anti-F4/80 (1:100, Miltenyi Biotech, 130-102-379), anti-arginase-1 (1:100, Santa Cruz, SC-18354), anti-Mac3 (1:100, BD Pharmingen, 550292), anti-CD3 (1:100, Abcam, ab11089), anti-CD8 (1:100, Cell Signaling, 98941), or anti-neutrophil (1:100, Cedariane, CL8993B) antibody overnight at 4 °C. For cell culture, cells were fixed with 4% paraformaldehyde for 10 min and incubated with anti-CD11b (1:100, BioLegend, 101206), anti-arginase-1 (1:100, Santa Cruz, sc-18354), or anti-iNOS (Abcam, ab3523) antibody. Sections were stained with Alexa Fluor^®^ 488-conjugated, 568-conjugated, and 647-conjugated appropriate secondary IgGs (1:500, Life Technologies) for 1 h at room temperature. Images were acquired with an AxioImager fluorescence microscope (Zeiss) equipped with AxioCam 506 CCD camera (Zeiss). For histological study, sections were stained with hematoxylin and eosin (H&E), and imaged with an AxioLab microscope (Zeiss) equipped with AxioCam HRC CCD camera (Zeiss).

### Brain tissue preparation and 3-D confocal imaging

The *Rosa-LSL-tdTomato*;*Tie2-Cre* mice bearing RCAS-induced tumors were perfused with 30 ml PBS and 10 ml 4% paraformaldehyde. The brains were dissected and fixed in 4% paraformaldehyde for 24 h at 4 °C. Hydrogels were prepared by using 40% acrylamide and 10% VA-044 in PBS. The brain was soaked in the hydrogel for 24 h at 4 °C, followed by polymerization in hydrogel at 37 °C for 3 h. The brains were cut into 2-mm-thick slices. The brain slices were placed in a holder and cleared for 3 h by electrophoretic tissue clearing with the X-CLARITY Tissue Clearing system (Logos Biosystems, C10001). The cleared brain slices were washed in PBS overnight, and incubated with FITC-conjugated anti-F4/80 antibody (1:100) and Alexa Fluor 647-conjugated anti-CD206 antibody (1:100) in PBS for 1 day at 37 °C. After washing with PBS for 1 day, the brain slices were incubated in the X-CLARITY mounting solution (Logos Biosystems, C13101) at 37 °C for 1 day. The slices were placed on the glass slide for confocal imaging with an SP8 laser microscope (Leica) in the Microscopy Core of the University of Pennsylvania. 3-D images were generated by using the Velocity software.

### Immunoblot analysis

Cells and tissues were lysed with a NP-40 buffer containing protease inhibitor cocktail (Roche, 11697498001). Protein (20–50 μg0 was resolved by 4–15% precast sodium dodecyl-sulfate polyacrylamide gel electrophoresis gel (Bio-Rad). After transfer, PVDF membranes were blotted with anti-GAPDH (1;1,000, Cell Signaling, 5174), anti-PCNA (1:1,000, Origene, TA800875), anti-mouse arginase-1 (1:1,000, Santa Cruz, sc-18354), anti-human IL-6 (1:1,000, GeneTex, GTX110527), anti-mouse IL-6 (1:1,000, Cell Signaling, 12912), anti-α-tubulin (1:1,000, Cell Signaling, 2144), anti-human CSF-1 (1:1,000, Abcam, ab9693), anti-HIF-1α (1:1,000, Cayman, 10006421), anti-HIF-2α (1:1,000, Origene, TA309641), anti-PPARγ (1:1,000, Cell Signaling, 2443), anti-P-Akt-Ser^473^ (1:1,000, Alzforum, AB3132), anti-Akt (1:1,000, Cell Signaling, 4685), anti-P-ERK1/2-Thr^202^/Tyr^204^ (1:1,000, Cell Signaling, 4370), anti-ERK1/2 (1:1,000, Cell Signaling, 4695), anti-P-mTOR-Ser^2448^ (1:1,000, Cell Signaling, 5536), anti-mTOR (1:1,000, Cell Signaling, 2983), or anti-HSP90 (1:1,000, Cell Signaling, 4874) antibody. Proteins were detected with horseradish peroxidase-conjugated antibodies specific for either rabbit or mouse IgG (Bio-Rad), followed by ECL development (GE Healthcare, RPN2232). Band density was quantified by using NIH Image J program. The uncropped blots were included as in Supplementary Fig. 19.

### Flow cytometry

Mouse glioma and normal brain tissues were isolated and subjected to mechanical dissociation with a gentleMACS Dissociator (Miltenyi Biotech) and enzymatic digestion with collagenase II and dispase II. The single-cell suspensions were prepared from mouse tumors and brains, BMDMs cells, and human BMDMs with or without co-culture with ECs. A total of 2 × 10^6^ cells were probed with anti-CD11b (1:100, BioLegend, 101206), anti-CD86 (1:100, BD Pharmingen, 553692), anti-CD206 (1:100, BioLegend,141708), anti-mouse IL-10 (1:100, BioLegend, 505009), anti-mouse IL-12 (1:100, BioLegend, 505203), or anti-human IL-10 (1:100, BioLegend, 506807) antibody with conjugation with different fluorescence dyes, or control IgG. The cells were analyzed with an Accuri C6 flow cytometer (BD Biosciences) by using FlowJo software.

### Statistical analysis

Student’s *t* test (unpaired, two-tailed) and log-rank (Mantel–Cox test) analysis were performed by using Prism software for statistical analysis between groups, and *p* < 0.05 was considered to represent a statistically significant difference.

### Data availability

All data are available within the Article and Supplementary Files, or available from the authors upon request.

## Electronic supplementary material

Supplementary Information

Description of Additional Supplementary Files

Supplementary Movie 1
