# Supplementary material for: Vascular niche IL-6 induces alternative macrophage activation in glioblastoma through HIF-2α
Source: Nat Commun. 2018 Feb 8;9:559. doi: 10.1038/s41467-018-03050-0 (PMC5805734; doi:10.1038/s41467-018-03050-0)
Supplement: Supplementary file 1 — Supplementary Information [file 41467_2018_3050_MOESM1_ESM.pdf]

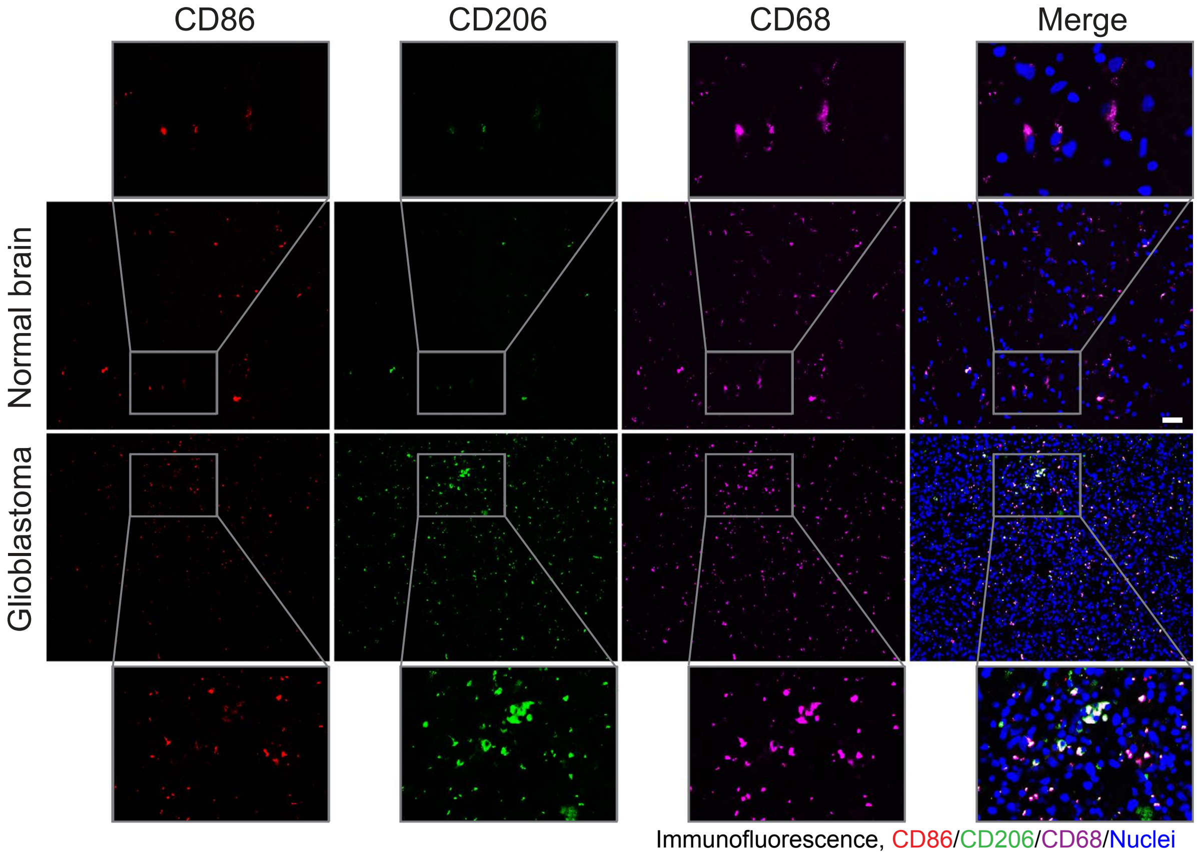

Supplementary Figure 1. CD86 and CD206 expression in normal brain- or GBM-associated CD68⁺ macroglias/ macrophages.
The brain sections from normal brain or surgical specimens of GBM tumors were probed with anti-CD86, anti-CD206, and anti-CD86 antibodies. Representative images are shown (n = 5 patients). Bar represents 100 μm. Zoom-in factor: 2.5.

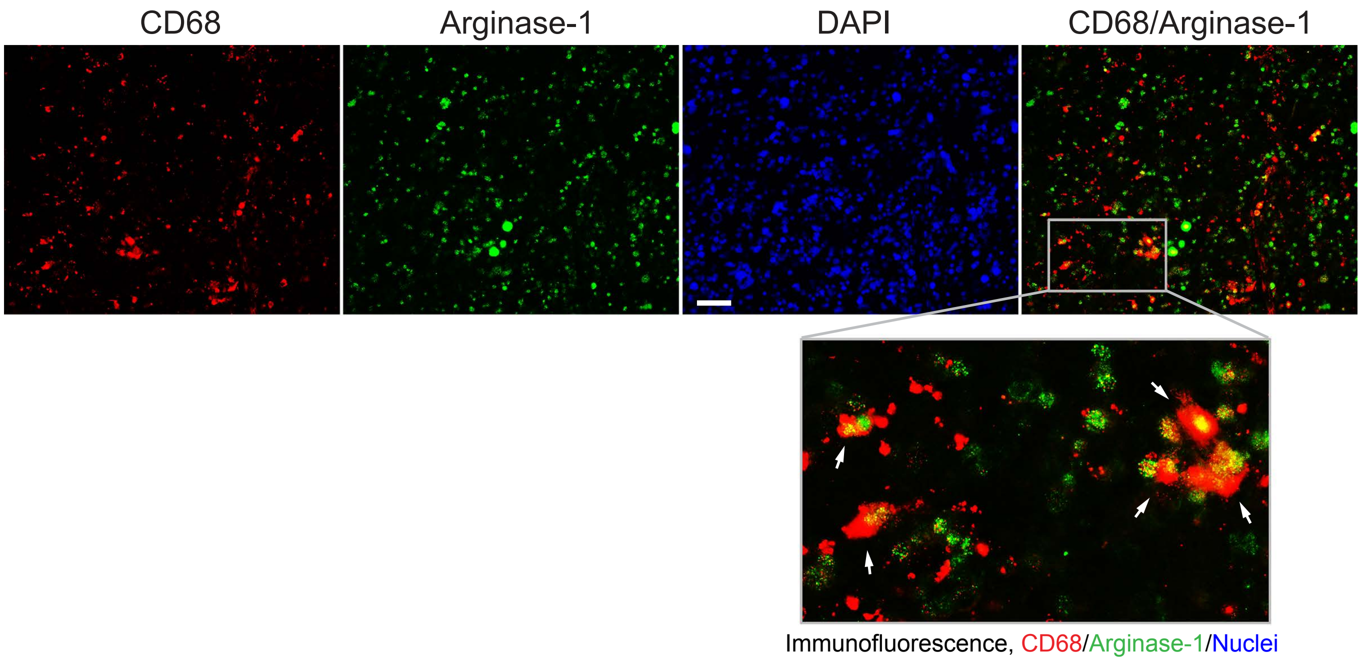

Supplementary Figure 2. GBM-associated CD68⁺ macrophages express arginase-1.
The sections from surgical specimens of GBM tumors were probed with anti-arginase-1, and anti-CD68 antibodies. Representative images are shown. $CD68^+$ and $CD68^+Arginase\text{-}1^+$ cells were counted. Quantitative data show that 76.2% ± 4.5% of $CD68^+$ cells are arginase-1^+ (n = 6 patients, mean ± SEM). Bar represents 100 μm. Zoom-in factor: 3.8.

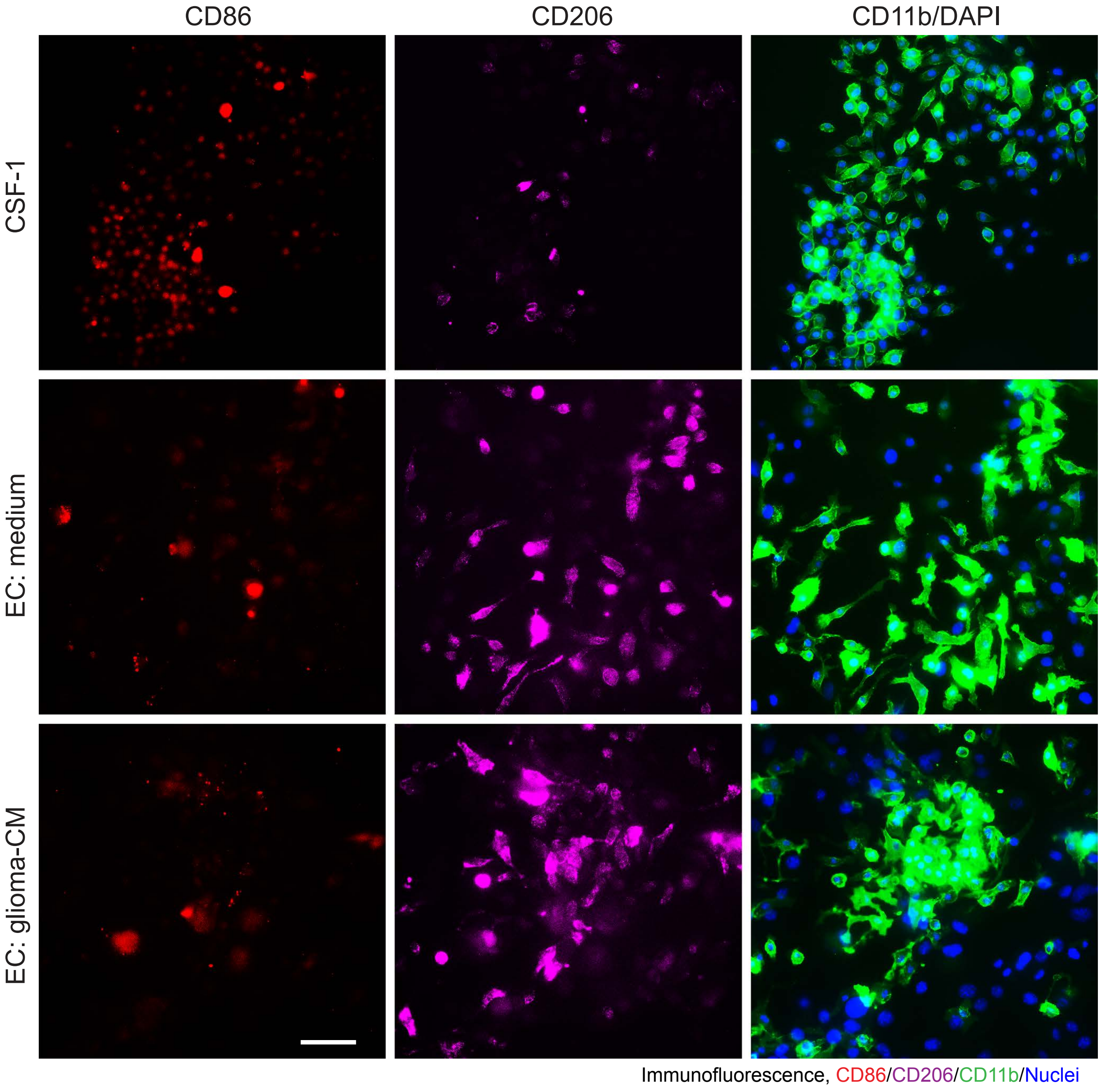

Supplementary Figure 3. Co-culture of BM-derived macrophages with ECs induces CD206 expression in macrophages. Mouse brain ECs were pre-treated with the glioam-conditioned medium (glioma-CM, harvested from medium supernatant of mouse GL26 glioma cells under 1% hypoxia) or control medium for 24 hr. Mouse bone marrow (BM)-derived macrophages were prepared by flushing cells from femur and tibia bones, and treated with CSF-1. BM-derived macrophages were incubated with CSF-1 or co-cultured with pretreated ECs for 5 days, stained with anti-CD11b, -CD86, -CD206 antibodies, and analyzed by immunofluorescence. Representative images are shown. Bar represents 100 μm.

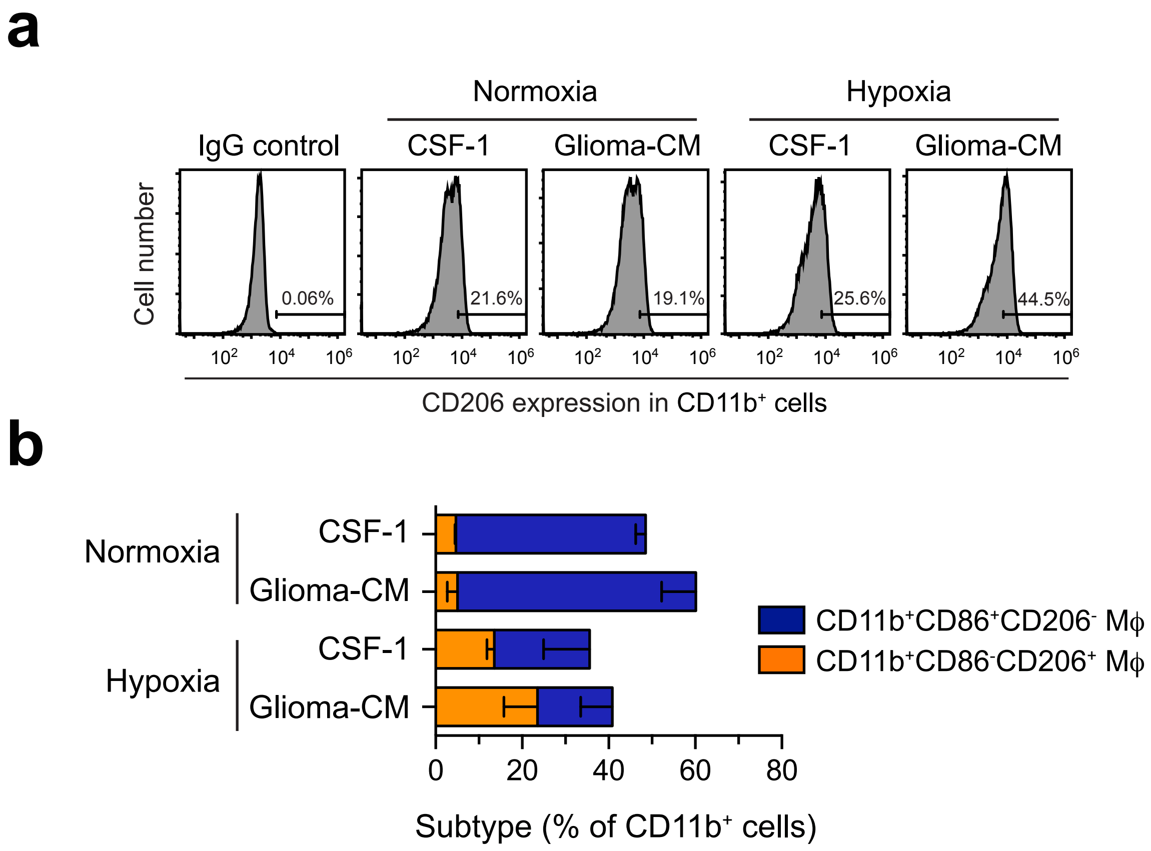

Supplementary Figure 4. Effects of glioma-CM and hypoxia on macrophage alternative activation.
Human peripheral blood mononuclear cell (PBMC)-derived monocytes were incubated with 10 ng/ml CSF-1 or glioma-CM under normoxia or hypoxia for 5 days, stained with anti-CD11b, anti-CD86, anti-CD206 antibodies, and analyzed by flow cytometry. (**a**) Representative results for CD206 expression in $CD11b^+$ cells. (**b**) Quantified data in sorted $CD11b^+$ macrophages (n = 3 independent experiments, mean ± SEM).

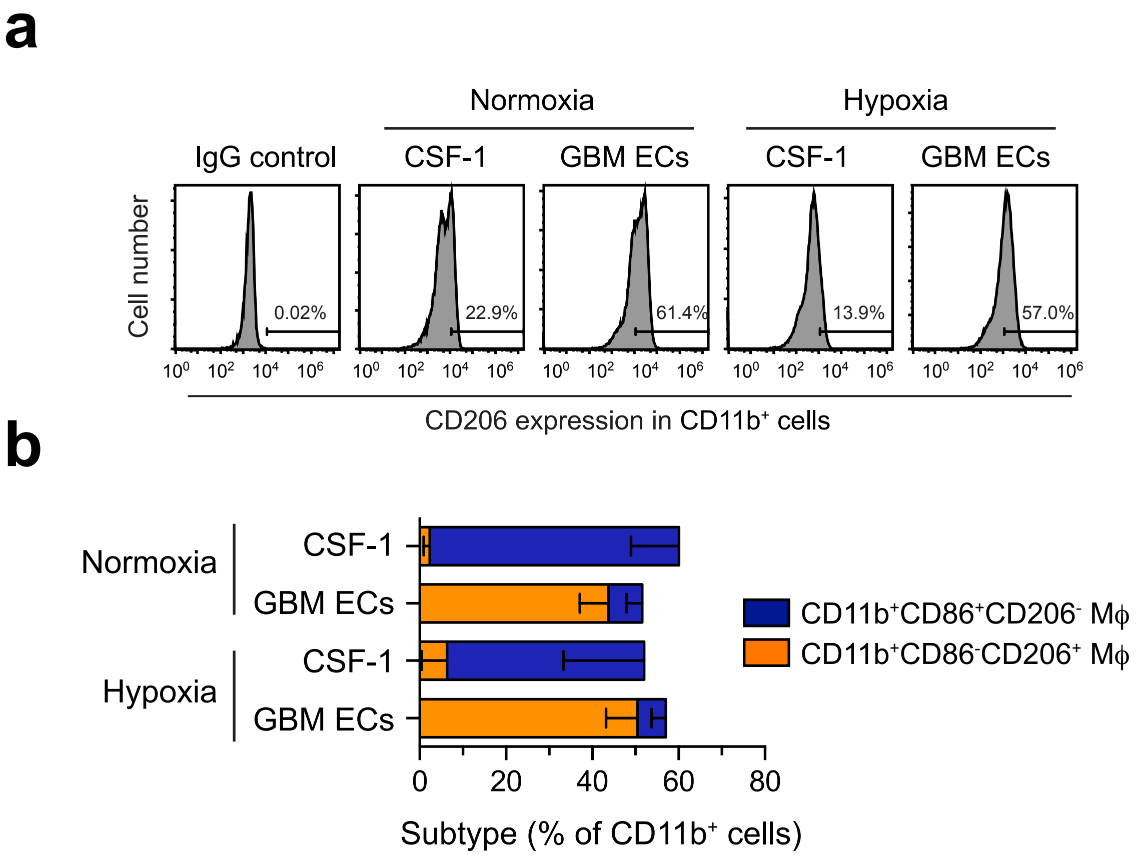

Supplementary Figure 5. Effects of hypoxia on EC-mediated macrophage alternative activation. Human peripheral blood mononuclear cell (PBMC)-derived monocytes were incubated with 10 ng/ml CSF-1 or co-cultured with tumor-associated ECs isolated from GBM patient #5377 tumors under normoxia or hypoxia for 5 days, stained with anti-CD11b, anti-CD86, anti-CD206 antibodies, and analyzed by flow cytometry. (**a**) Representative results for CD206 expression in CD11b$^+$ cells. (**b**) Quantified data in sorted CD11b$^+$ macrophages (n = 4 independent experiments, mean ± SEM).

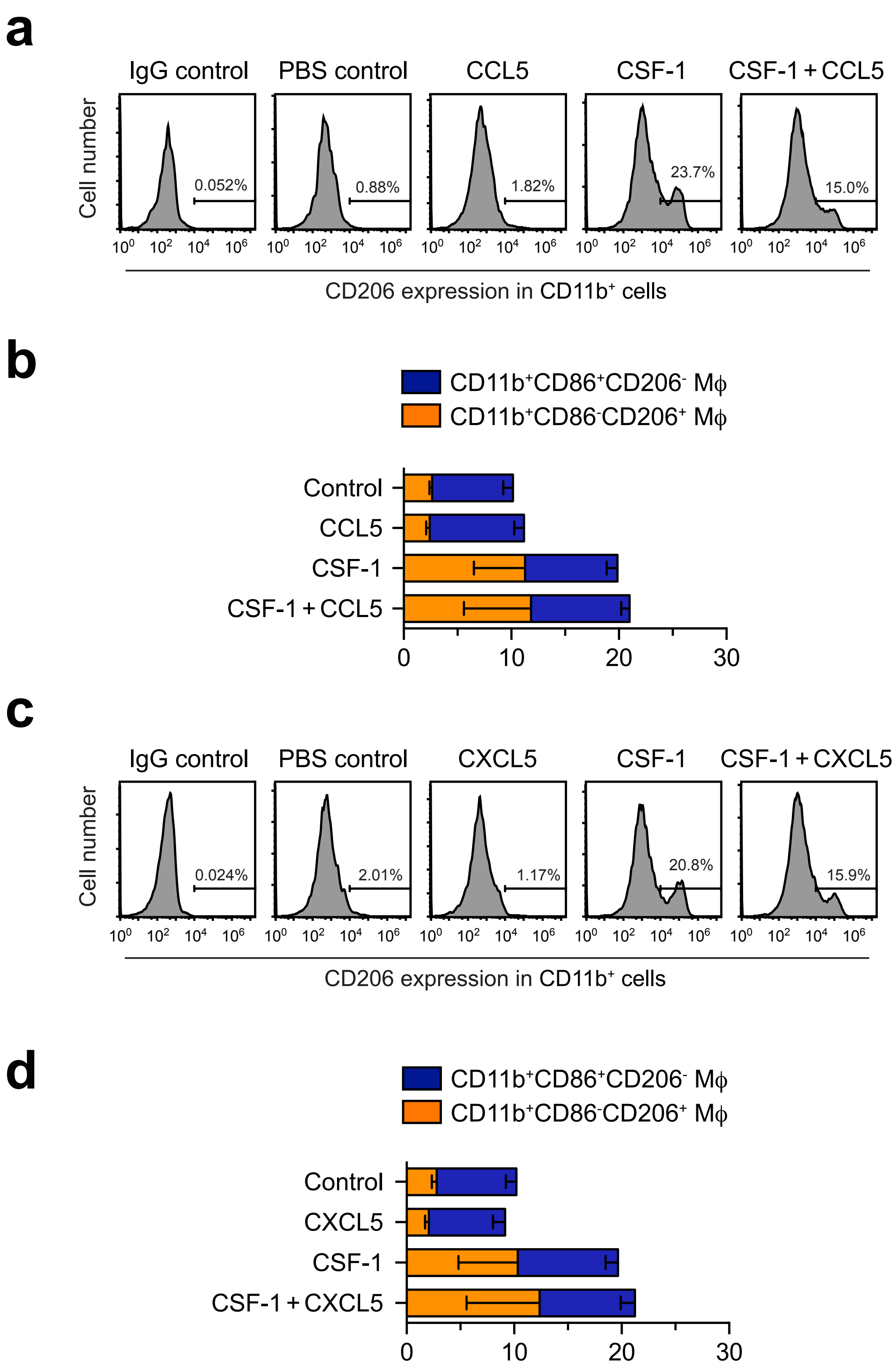

Supplementary Figure 6. Effects of CCL5 and CXCL5 on macrophage alternative activation.
Mouse bone marrow (BM)-derived macrophages were incubated with 10 ng/ml CSF-1 and 100 ng/ml CCL5 or CXCL5 for 5 days, stained with anti-CD11b, anti-CD86, anti-CD206 antibodies, and analyzed by flow cytometry. (**a**) Representative results for CD206 expression in CCL5-treated CD11b$^+$ cells. (**b**) Quantified data in sorted CCL5-treated CD11b$^+$ macrophages (Mφ, n = 6, mean ± SEM). (**c**) Representative results for CD206 expression in CXCL5-treated CD11b$^+$ cells. (**d**) Quantified data in sorted CXCL5-treated CD11b$^+$ macrophages (n = 6, mean ± SEM).

a

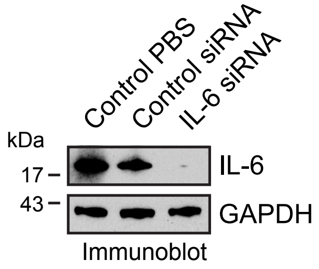

b

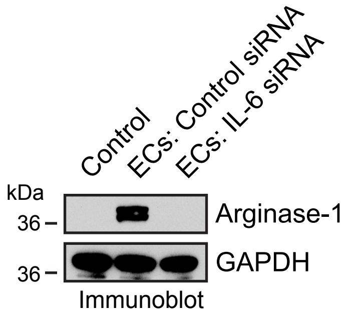

Supplementary Figure 7. Effects of IL-6 knockdown on EC-induced arginase-1 expression.
Mouse ECs were treated with glioma-CM and transfected with control or IL-6 siRNA. Mouse BM-derived macrophages were incubated with EC-conditioned medium for 5 days. Cell lysates from (**a**) ECs and (**b**) macrophages were immunoblotted.

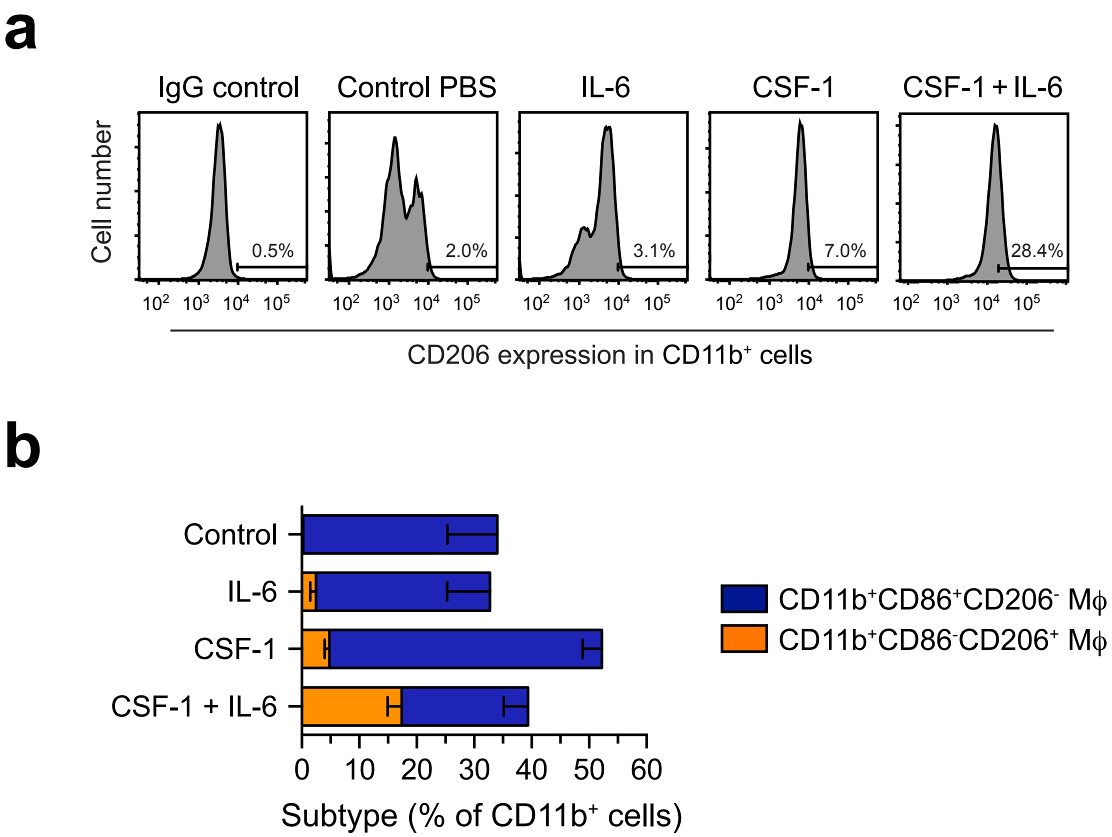

Supplementary Figure 8. Effects of IL-6 and CSF-1 on human macrophage M2 activation.
Human peripheral blood mononuclear cell (PBMC)-derived monocytes were incubated with 10 ng/ml CSF-1 and co-cultured with tumor-associated ECs isolated from GBM patient #5377 tumors under normoxia or hypoxia for 5 days, stained with anti-CD11b, anti-CD86, anti-CD206 antibodies, and analyzed by flow cytometry. (**a**) Representative results CD206 expression in $CD11b^+$ cells. (**b**) Quantified data in sorted $CD11b^+$ macrophages (Mφ, n = 4 independent experiments, mean ± SEM).

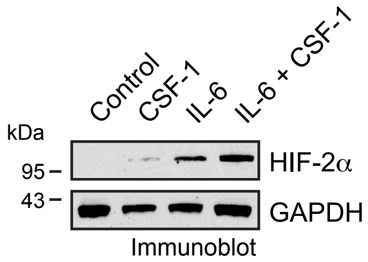

Supplementary Figure 9. Effects of IL-6 and CSF-1 on HIF-2α expression in human monocytes.
Human peripheral blood mononuclear cell (PBMC)-derived monocytes were incubated with 10/ml ng CSF-1 and 100 ng/ml IL-6 for 5 days. Cells were lysed and subjected to immunoblot analysis.

a

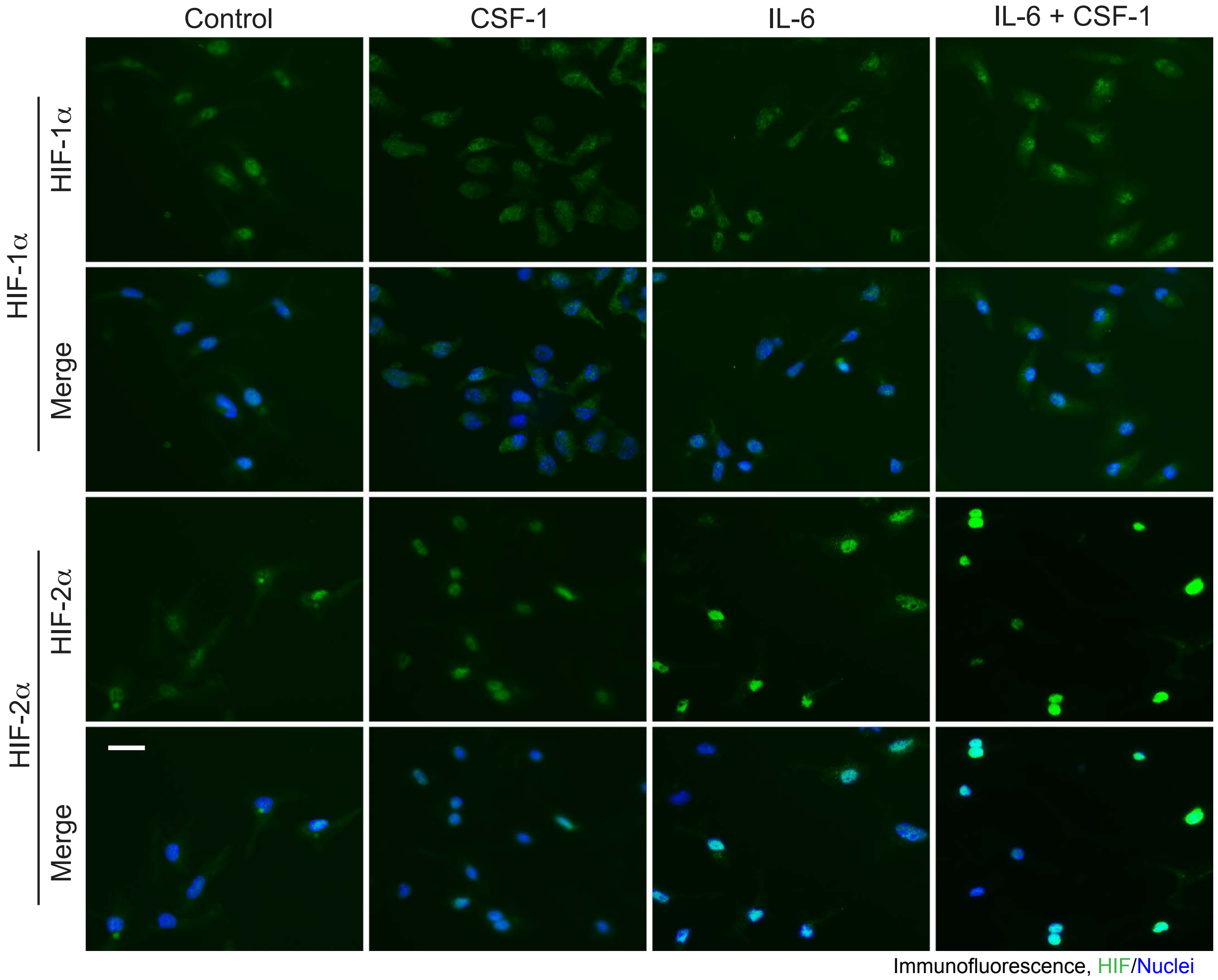

b

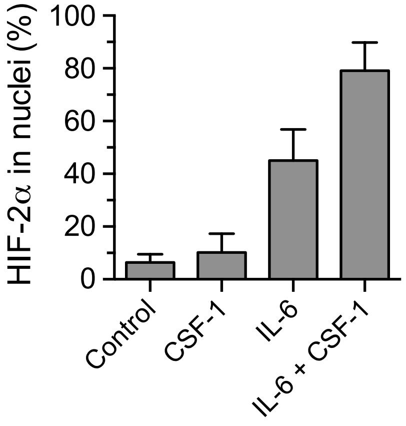

Supplementary Figure 10. IL-6 and CSF-1 induce HIF-2α translocation to nuclei.
Mouse bone marrow (BM)-derived macrophages were incubated with 10/ml ng CSF-1 and 100 ng/ml IL-6 for 5 days, stained with anti-HIF-1α and anti-HIF-2α antibodies, and analyzed by immunofluorescence. (a) Representative images are shown. (b) Cells with nuclei translocation of HIF-2α were counted (n = 6, mean ± SEM). Bar represents 20 μm.

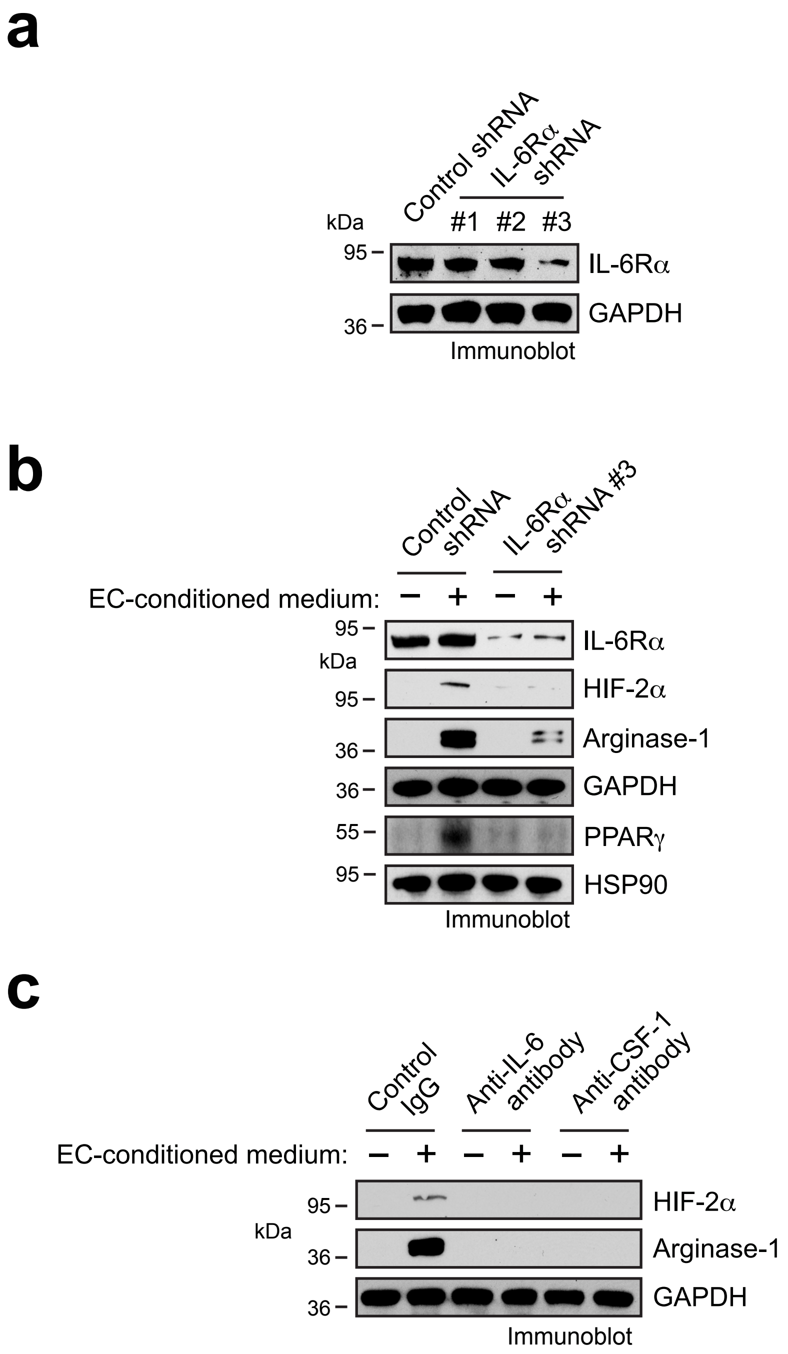

Supplementary Figure 11. Effects of IL-6 receptor knockdown and IL-6/CSF-1 neutralization on EC-induced HIF-2α and arginase-1 expression.

Mouse ECs were treated with mouse glioma-conditioned medium. (**a**, **b**) Mouse BM-derived macrophages were lentivirally transduced with shRNA targeting IL-6 receptor-α (IL-6Rα). (**a**) Mouse macrophages were lyzed and immunoblotted. (**b**) Macrophages were treated with EC-conditioned medium for 5 days, and subjected to immunoblot analysis. (**c**) Mouse BM-derived macrophages were incubated with EC-conditioned medium for 5 days in the presence of control IgG, anti-IL-6 antibody, or anti-CSF-1 antibody. Cells were lysed and subjected to immunoblot analysis.

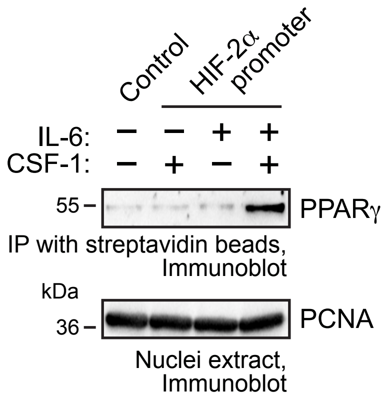

Supplementary Figure 12. Cotreatment with IL-6 and CSF-1 induce PPARγ binding to HIF-2α promoter.
Mouse BM-derived macrophages were treated with or without CSF-1 and IL-6 for 3 days. Nuclei protein was incubated with biotin-labeled synthetic DNAs that encode control scrambled or HIF-2α promoter sequence, followed by immunoprecipitation with streptavidin-conjugated beads. Precipitants and nuclei protein were immunoblotted.

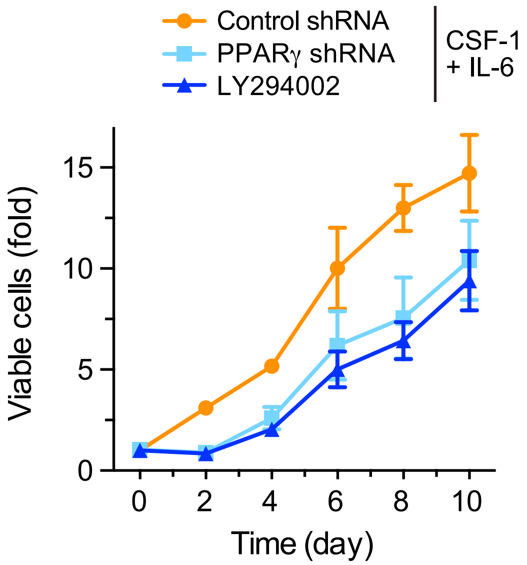

Supplementary Figure 13. Effects of IL-6 and CSF-1 on HIF-2α and arginase-1 expression.
Mouse BM-derived macrophages were pretreatment with 1 μM LY294002, or lentivirally transduced to express shRNA targeting PPARγ or scrambled sequence. Cells were incubated with 10/ml ng CSF-1 and 100 ng/ml IL-6 for 5 days. Cell viability was determined (n = 3, mean ± SEM).

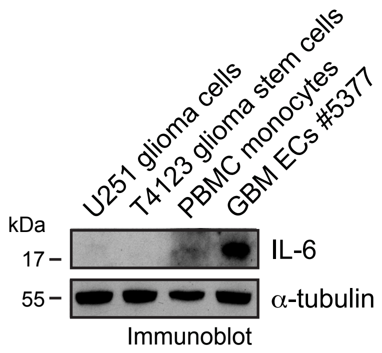

Supplementary Figure 14. IL-6 expression in different cell types.
Different cells were lysed and subjected to immunoblot analysis.

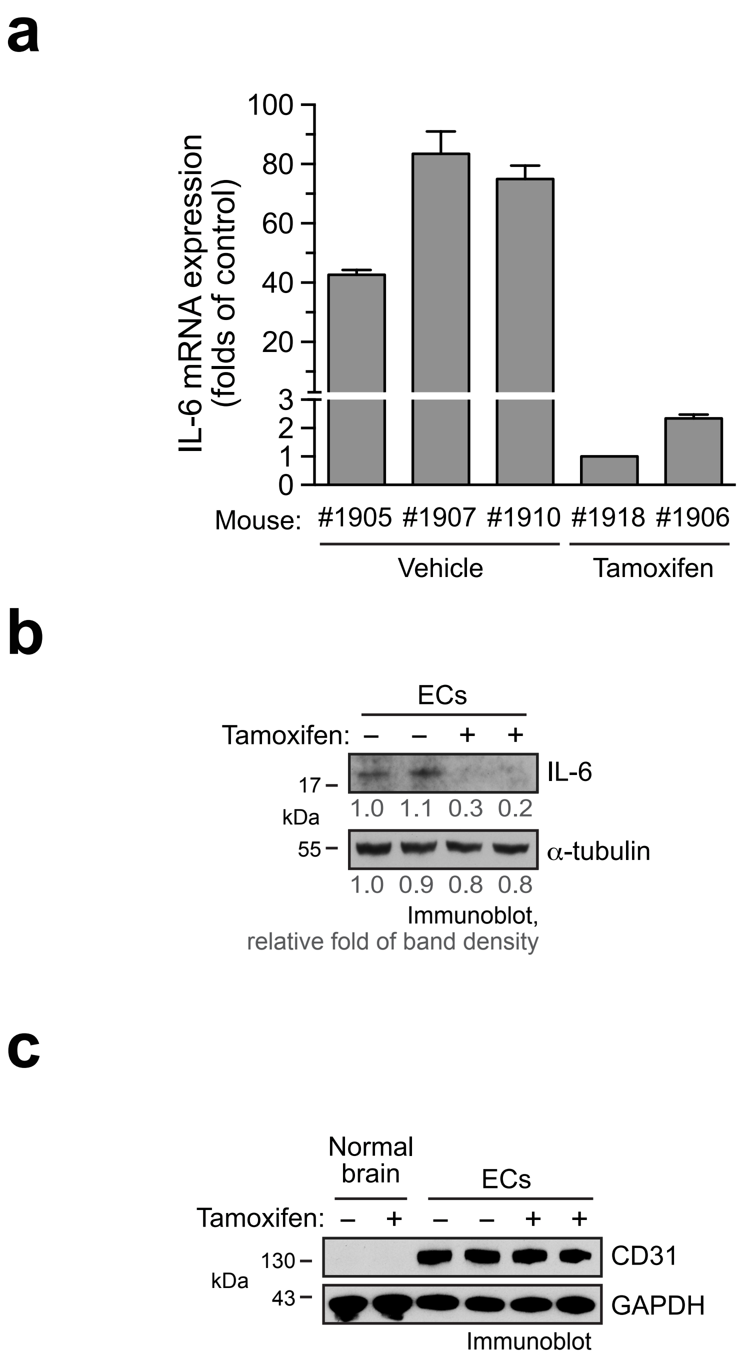

Supplementary Figure 15. EC-specific IL-6 knockout.
Cdh5-Cre^{ERT2}*;Il6*$^{fl/fl}$ mice were injected with tamoxifen. Brain microvascular ECs were isolated. (**a**) Quantitative RT-PCR analysis of IL-6 mRNA expression. Results were normalized to GAPDH levels (n = 3, mean ± SEM). (**b**) Brain ECs were lysed and immunoblotted. Band density was quantified. (**c**) Normal brain tissues and ECs were lysed and subjected to immunoblot analysis.

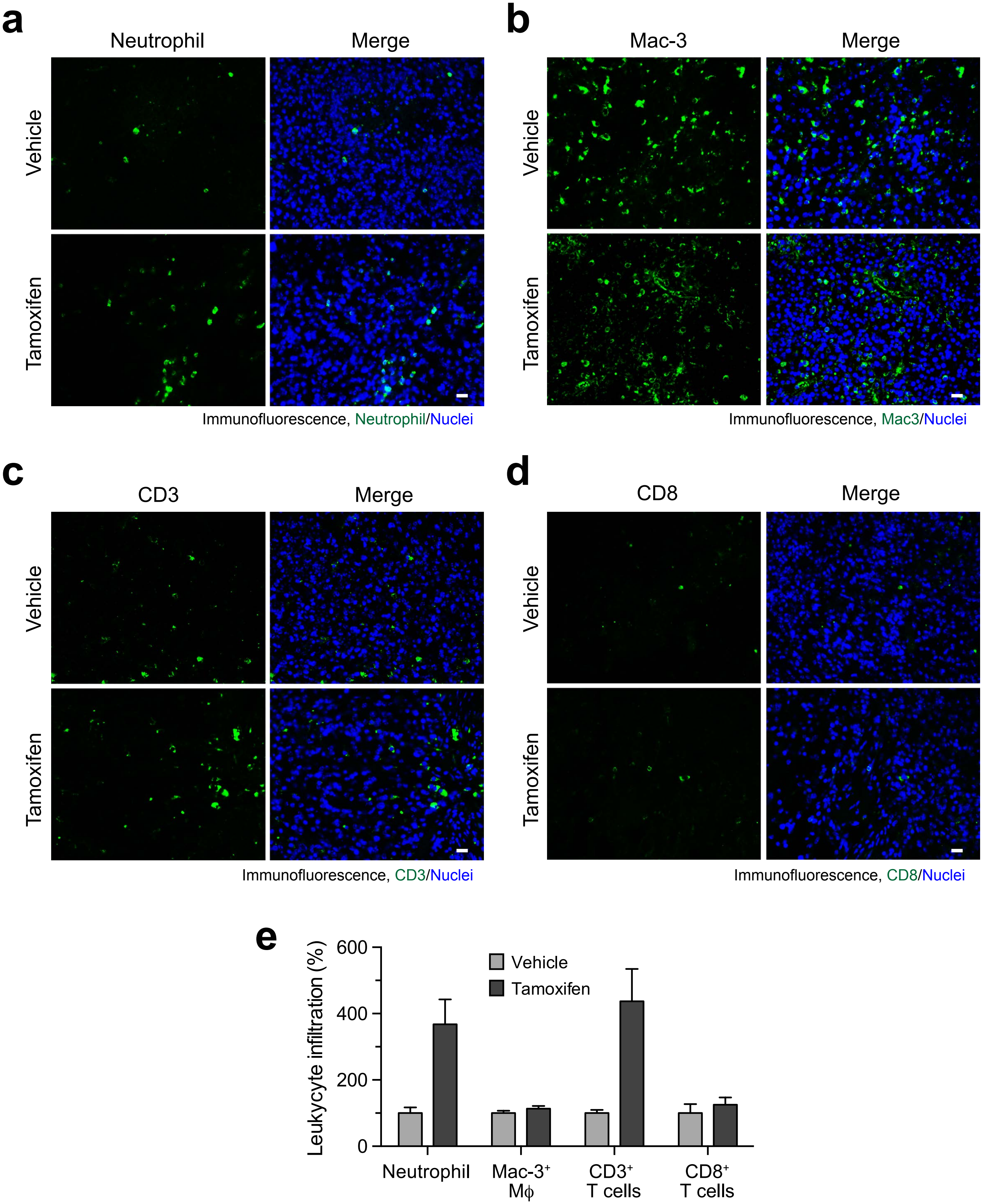

Supplementary Figure 16. Leukocyte infiltration in mouse glioma.
Cdh5-Cre^{ERT2};*Il6*$^{fl/fl}$ mice were injected with tamoxifen and implanted with glioma cells. Tumor sections were stained with different antibodies and imaged. (**a**-**d**) Representative images. Bar represents 20 μm. (**e**) Quantified results (n = 3 mice, mean ± SD).

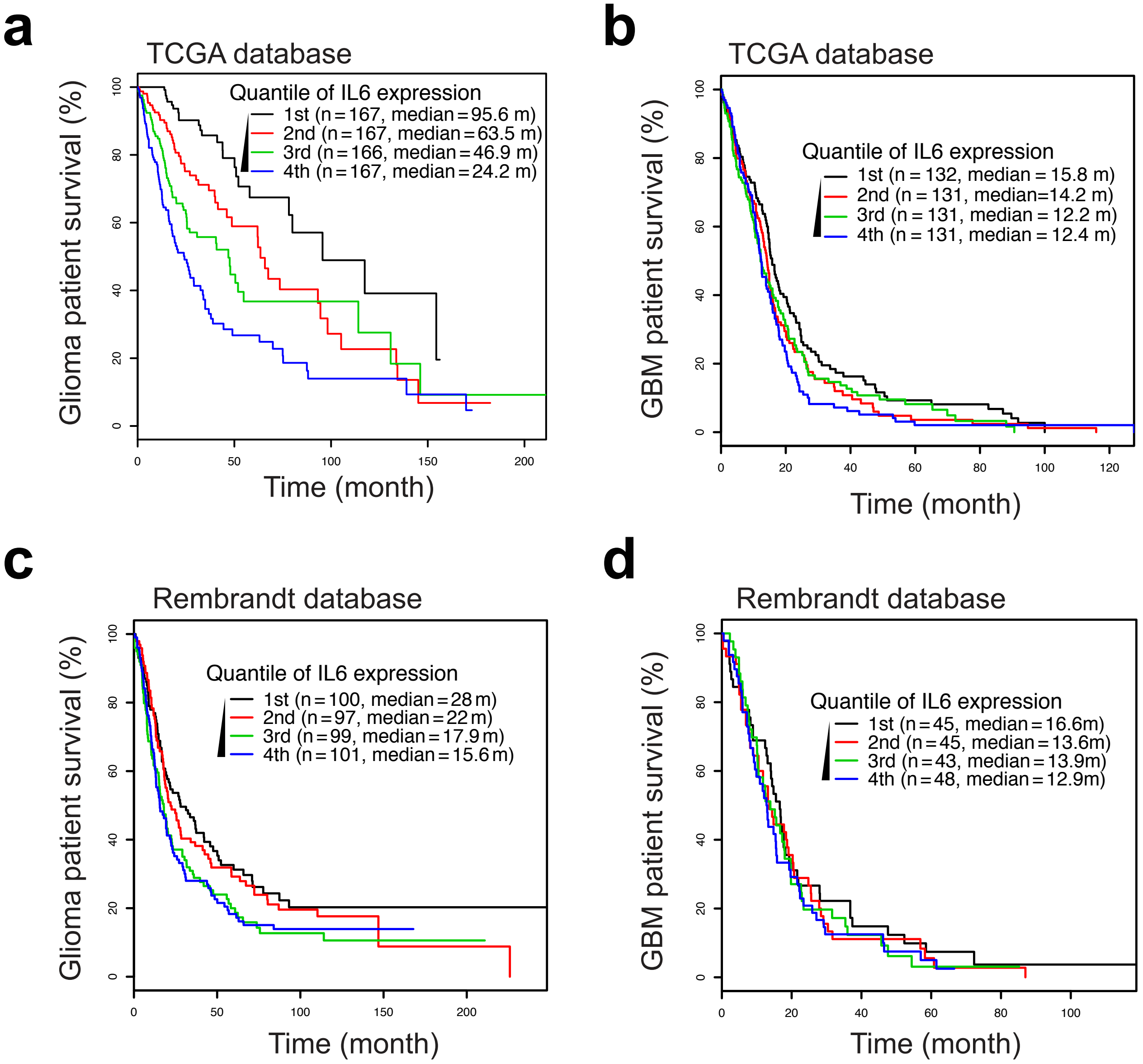

Supplementary Figure 17. High IL-6 expression correlates with poor survival in glioma patients.
IL-6 mRNA expression and patient overall survival were analyzed in TCGA and Rembrandt databeses in glioma and GBM patients.

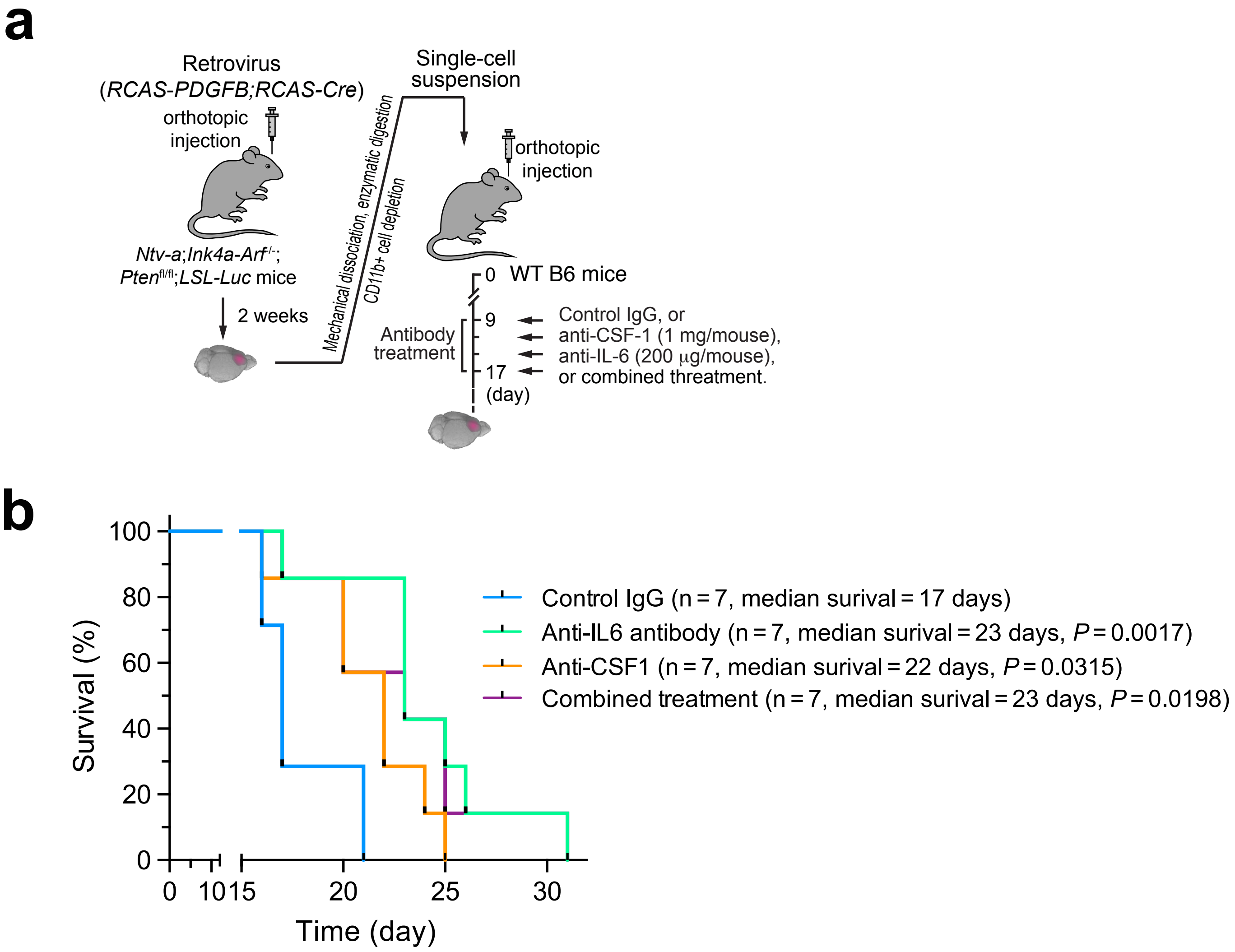

Supplementary Figure 18. Experimental therapy by anti-IL6 and anti-CSF1 neutralization.
GBM was induced in mice, followed by treatment with control IgG, anti-IL-6, and anti-CSF-1 antibody. (**a**) Experimental approach. (**b**) Animal survival was monitored. *P* values were determined by LogRank test.

Fig. 2d

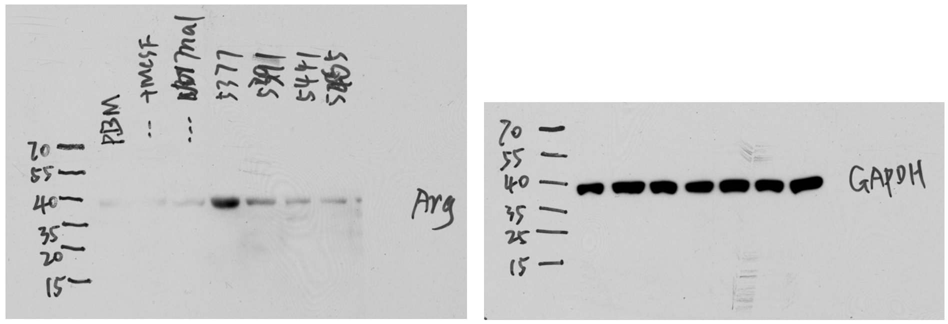

Fig. 3b

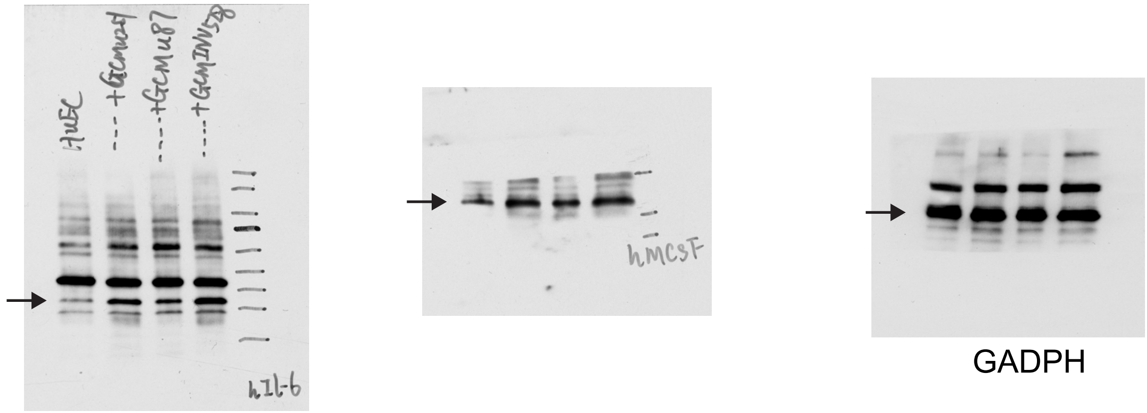

Fig. 3c

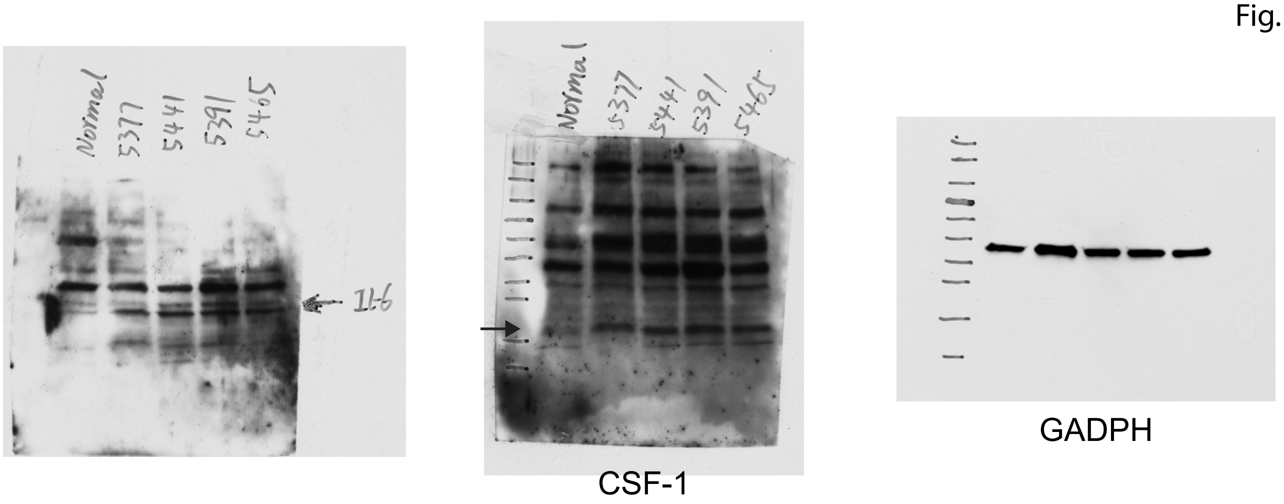

Fig. 3i

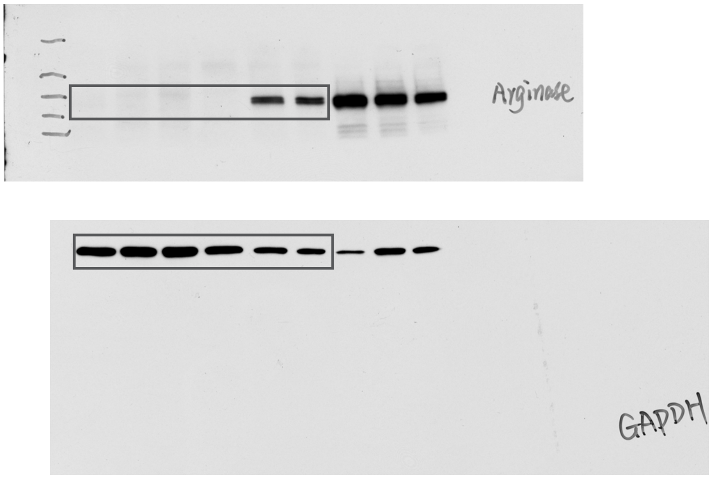

Supplementary Figure 19. Uncropped blots.

Fig. 4c

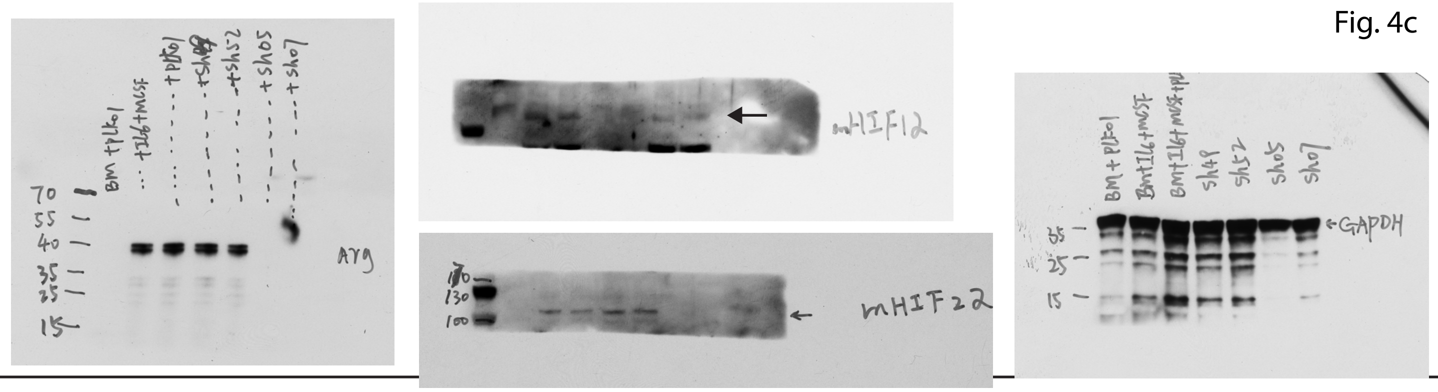

Fig. 5c

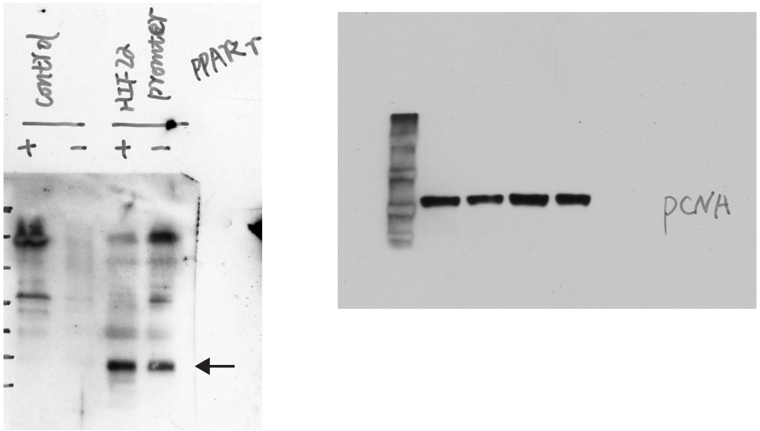

Fig. 5e

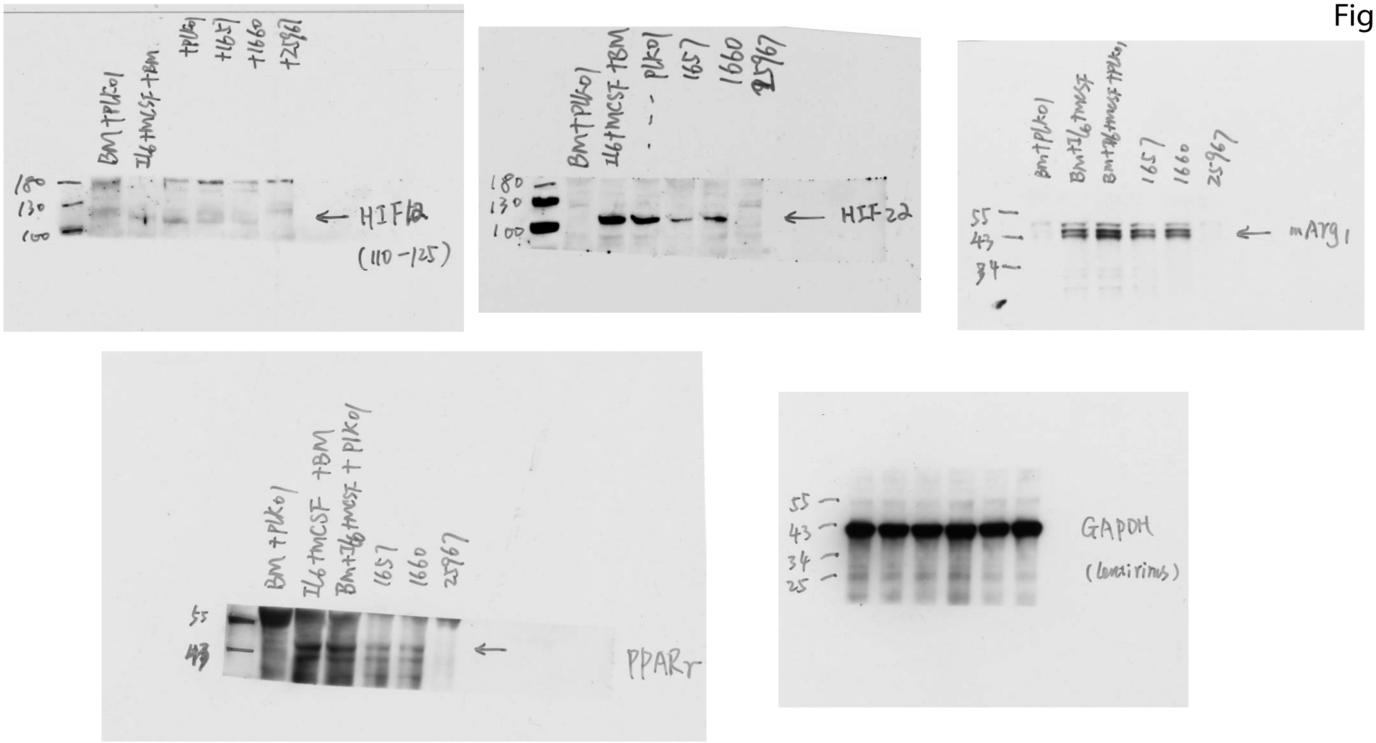

Supplementary Figure 19. Uncropped blots.

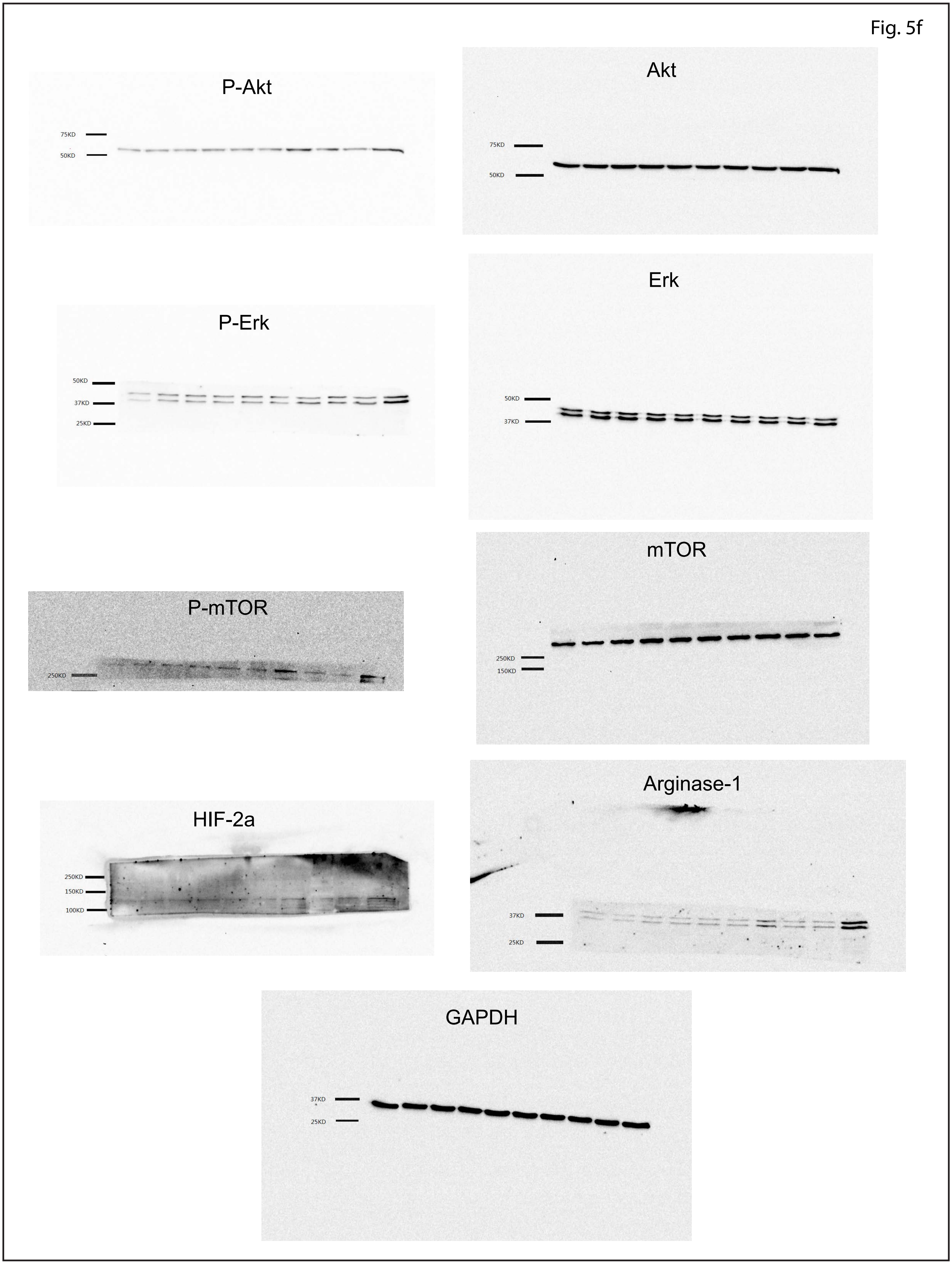

Supplementary Figure 19. Uncropped blots.

Fig. 6b

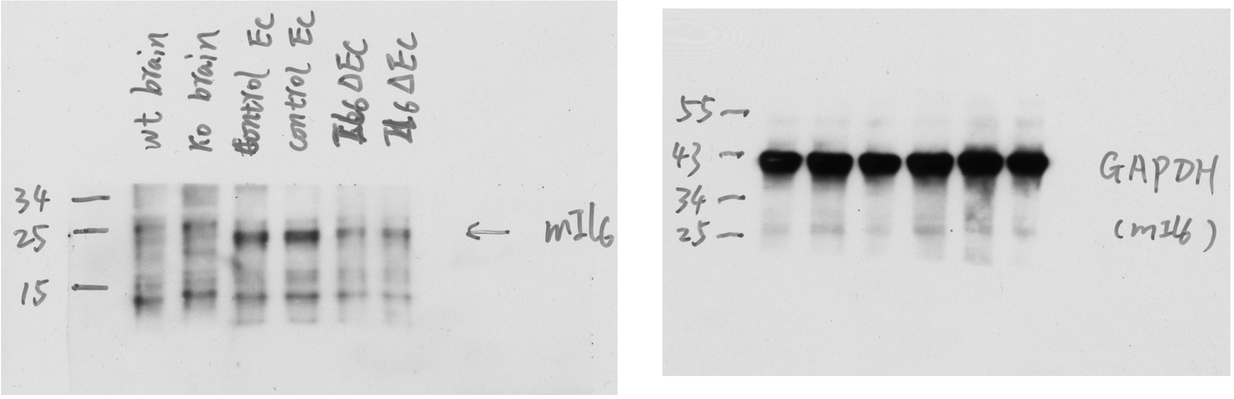

Fig. 6e

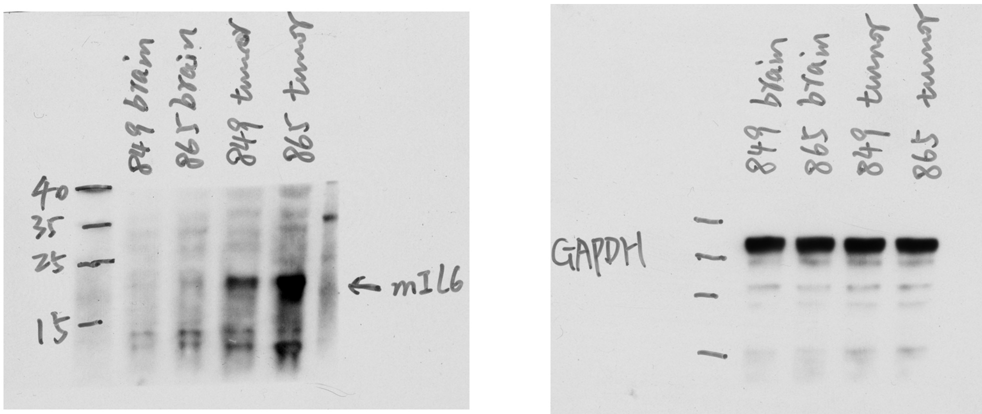

Supplementary Figure 19. Uncropped blots.

Suppl. Fig. 7a

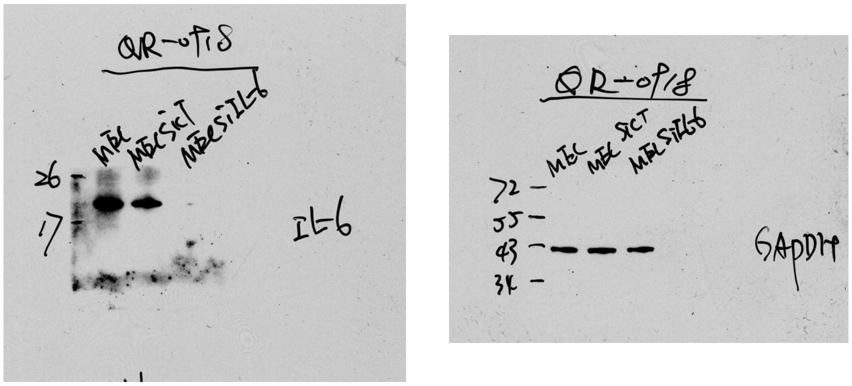

Suppl. Fig. 7b

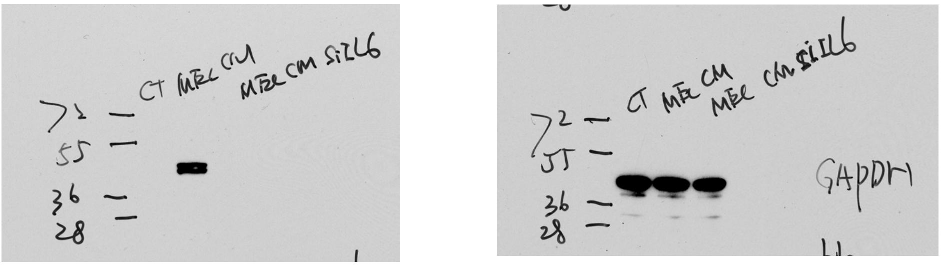

Suppl. Fig. 9

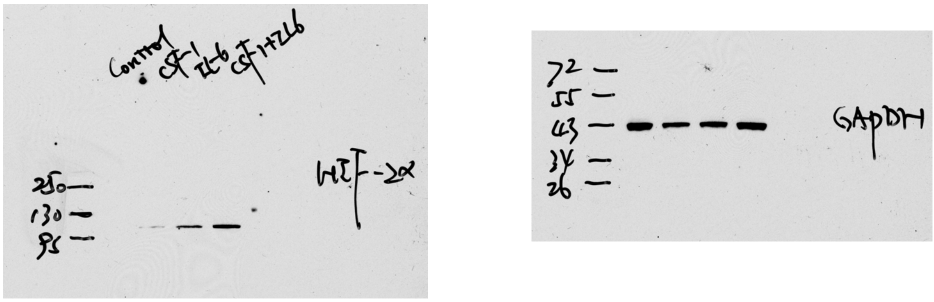

Suppl. Fig. 11a

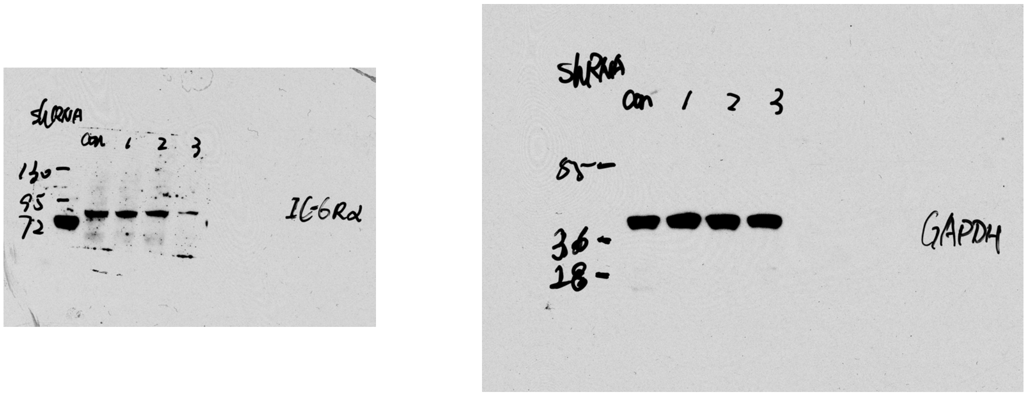

Supplementary Figure 19. Uncropped blots.

Suppl. Fig. 11b

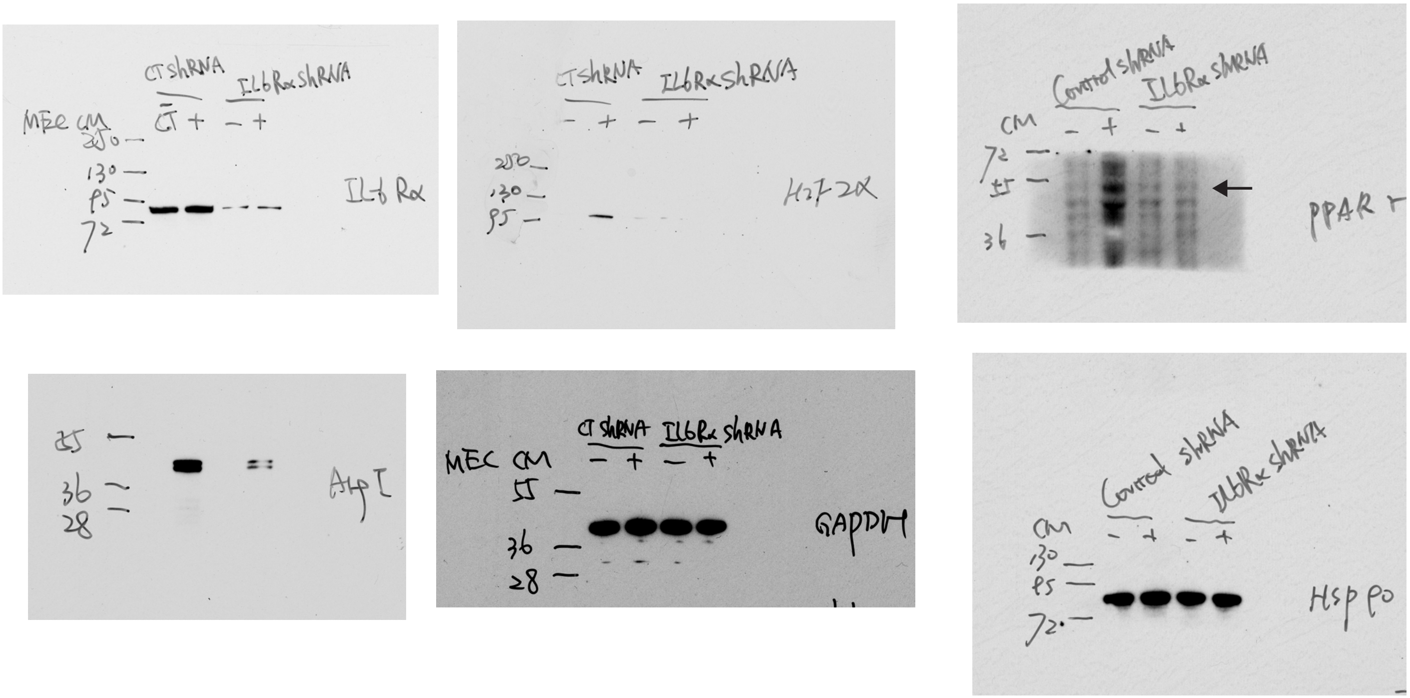

Suppl. Fig. 11c

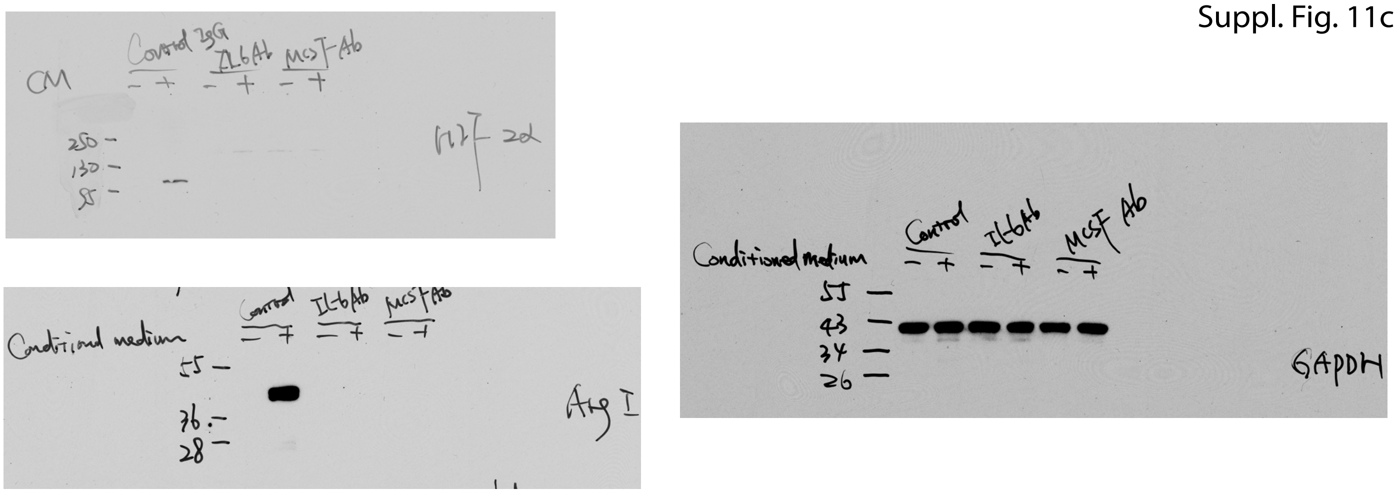

Suppl. Fig. 12

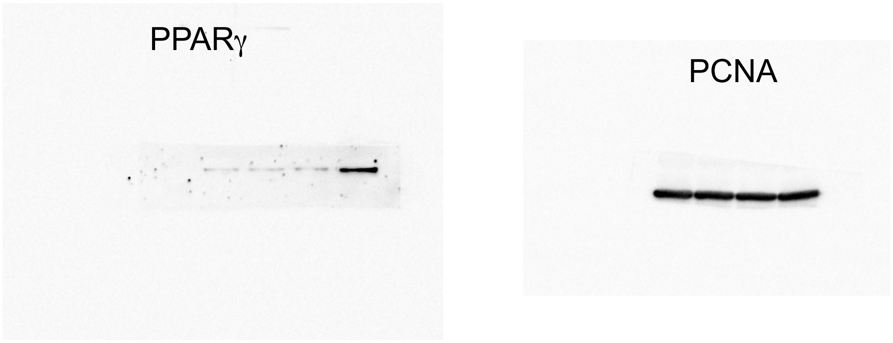

Supplementary Figure 19. Uncropped blots.

Suppl. Fig. 14

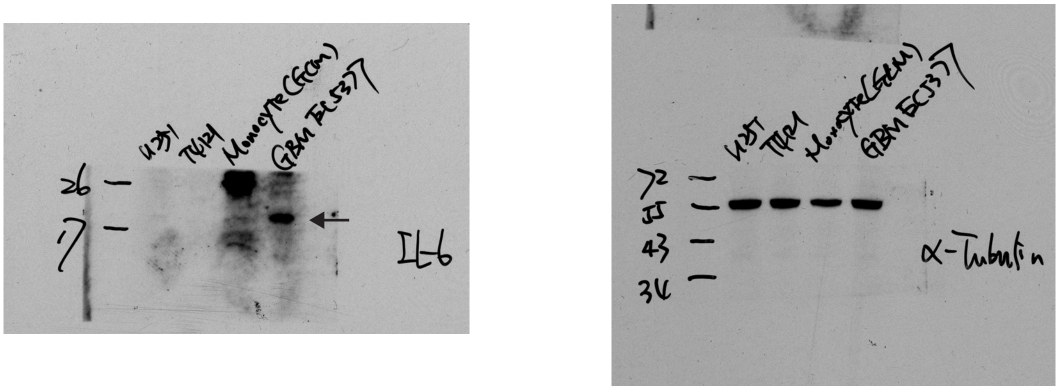

Suppl. Fig. 15b

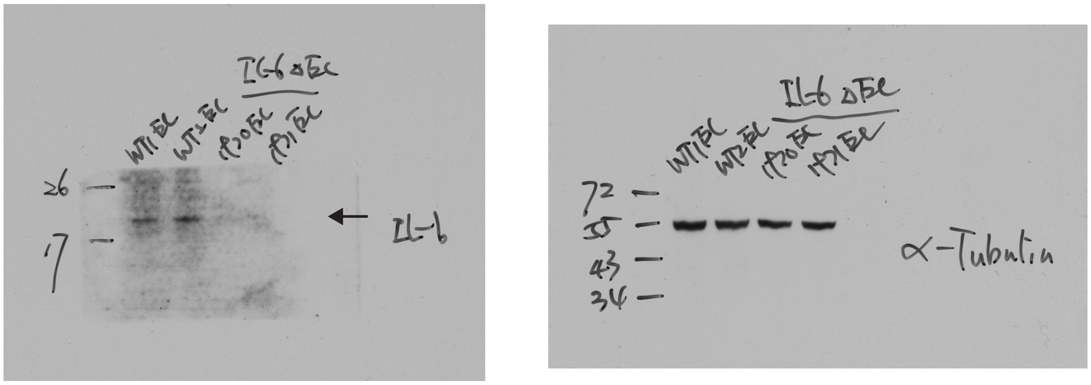

Suppl. Fig. 15c

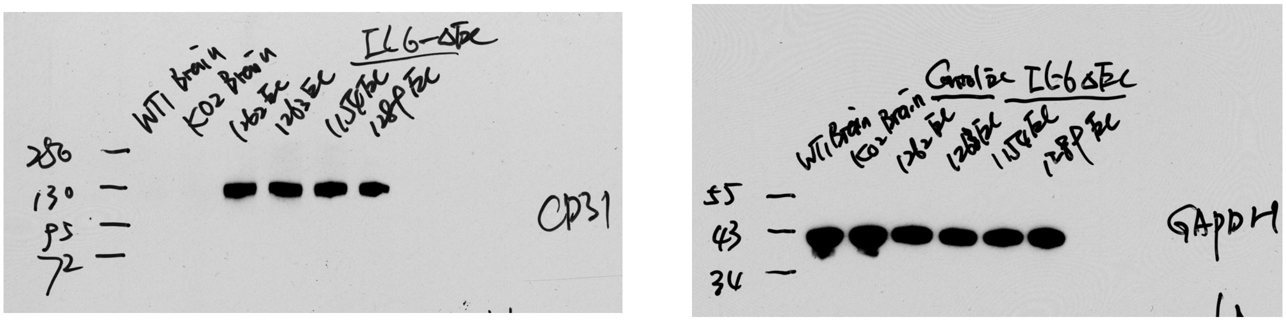

Supplementary Figure 19. Uncropped blots.
