# Supplementary material for: Vascular niche IL-6 induces alternative macrophage activation in glioblastoma through HIF-2α
Source: Nat Commun. 2018 Feb 8;9:559. doi: 10.1038/s41467-018-03050-0 (PMC5805734; doi:10.1038/s41467-018-03050-0)
Supplement: Supplementary file 2 — Description of Additional Supplementary Files [file 41467_2018_3050_MOESM2_ESM.pdf]

Description of Additional Supplementary Files

File Name: Supplementary Movie 1

Description: 3-D images of macrophages and vasculatures in GBM. GBM was induced by RCAS-mediated gene transfer in Ntv-a;Ink4a-Arf-/- ;Ptenfl/fl;LSL-Luc mice, followed by orthotopic tumor transplantation into Rosa-LSL-tdTomato;Tie2-Cre mice. Thick sections were stained with antiF4/80 and anti-CD206 antibodies, and subjected to confocal scanning imaging. 3-D images and rotation movie were generated by using velocity software. Blue, tdTomato-labeled vasculature; Greed, F4/80; Red, CD206.
